# The NtrYX Two-Component System of *Paracoccus denitrificans* Is Required for the Maintenance of Cellular Iron Homeostasis and for a Complete Denitrification under Iron-Limited Conditions

**DOI:** 10.3390/ijms23169172

**Published:** 2022-08-15

**Authors:** Alfonso Olaya-Abril, Víctor M. Luque-Almagro, Jesús Hidalgo-Carrillo, Eduardo Chicano-Gálvez, Francisco J. Urbano, Conrado Moreno-Vivián, David J. Richardson, María Dolores Roldán

**Affiliations:** 1 Departamento de Bioquímica y Biología Molecular, Universidad de Córdoba, Edificio Severo Ochoa, 1ª Planta, Campus de Rabanales, 14071 Córdoba, Spain; 2 Departamento de Química Orgánica, Instituto Universitario de Investigación en Química Fina y Nanoquímica (IUNAN), Universidad de Córdoba, Edificio Marie Curie, Campus de Rabanales, 14071 Córdoba, Spain; 3 IMIBIC Mass Spectrometry and Molecular Imaging Unit (IMSMI), Maimonides Biomedical Research Institute of Cordoba (IMIBIC), Reina Sofia University Hospital, University of Cordoba (UCO), 14004 Córdoba, Spain; 4 School of Biological Sciences, University of East Anglia, Norwich Research Park, Norwich NR4 7TJ, UK

**Keywords:** denitrification, ferric uptake regulator, iron homeostasis, nitrate reduction, nitrite reductase, nitrous oxide reductase, NtrYX system, *Paracoccus*

## Abstract

Denitrification consists of the sequential reduction of nitrate to nitrite, nitric oxide, nitrous oxide, and dinitrogen. Nitrous oxide escapes to the atmosphere, depending on copper availability and other environmental factors. Iron is also a key element because many proteins involved in denitrification contain iron-sulfur or heme centers. The NtrYX two-component regulatory system mediates the responses in a variety of metabolic processes, including denitrification. A quantitative proteomic analysis of a *Paracoccus denitrificans* NtrY mutant grown under denitrifying conditions revealed the induction of different TonB-dependent siderophore transporters and proteins related to iron homeostasis. This mutant showed lower intracellular iron content than the wild-type strain, and a reduced growth under denitrifying conditions in iron-limited media. Under iron-rich conditions, it releases higher concentrations of siderophores and displayes lower nitrous oxide reductase (NosZ) activity than the wild-type, thus leading to nitrous oxide emission. Bioinformatic and qRT-PCR analyses revealed that NtrYX is a global transcriptional regulatory system that responds to iron starvation and, in turn, controls expression of the iron-responsive regulators *fur*, *rirA*, and *iscR*, the denitrification regulators *fnrP* and *narR*, the nitric oxide-responsive regulator *nnrS*, and a wide set of genes, including the *cd*_1_-nitrite reductase NirS, nitrate/nitrite transporters and energy electron transport proteins.

## 1. Introduction

The prokaryotic *ntrYX* operon encodes the sensor-histidine kinase NtrY and response regulator NtrX. This NtrYX two-component system has been studied across the bacterial domain, and it has been generally associated with the regulation of nitrogen metabolism and, specifically, to nitrogen fixation, denitrification, and nitrate assimilation [1,2,3]. The *ntrYX* genes are located downstream of the *ntrBC* genes in many α-proteobacteria [3]. The NtrBC two-component regulatory system is composed of the nitrogen-sensor protein NtrB, which participates in the phosphorylation and activation, under nitrogen limiting conditions, of the DNA-binding protein NtrC, which, in turn, binds to a specific sequence in the promoter region of its targeted genes under nitrogen starvation [3,4,5]. The NtrYX system of *Brucella* spp. has been associated with redox sensing and denitrification, and a *ntrY* mutant exhibited a downregulated expression of the nitrate reductase (*narGHIJK*), nitrite reductase (*nirKV*), nitric oxide reductase (*norBCDEFQ*), and nitrous oxide reductase (*nosDFLRKYZ*) gene clusters under aerobic or microaerobic conditions [2,6,7]. Additionally, the NtrYX system of *Neisseria gonorrhoeae* has been postulated as a key regulator in the expression of respiratory enzymes, such as the nitrite and nitric oxide reductases and cytochrome *c* oxidase (CcoP subunit), and controlling biofilm formation and virulence [8]. In *Bradyrhizobium diazoefficiens*, the NtrYX system plays a key role in the symbiotic nitrogen fixation of soybean plants and *cbb*_3_ oxidase expression in bacteroides [9]. Recently, a role of the NtrYX system of *Rhodobacter sphaeroides* has been demonstrated in regulating the cell envelope, including peptidoglycan biosynthesis/modification and cell division [10]. Curiously, in several marine *Roseobacter* and *Ruegeria* species, the NtrYX system has been described to control the metabolism of ectoine (1,4,5,6-tetrahydro-2-methyl-4-pyrimidine carboxylic acid), which is a compatible solute, widely distributed among halophilic and halotolerant microorganisms, that prevents osmotic stress in highly saline environments [11]. 

α-proteobacterium *Paracoccus denitrificans* has been considered as a model for the mitochondria electron transport chain, which contains electron transport flavoproteins, NADH-ubiquinone oxidoreductase, *bc*_1_ complex, *c*-type cytochromes, and an *aa*_3_-type terminal cytochrome oxidase. Members of the genus exhibit a great range of metabolic flexibility, particularly with respect to processes involving respiration, such as the use in denitrification of nitrate, nitrite, nitrous oxide, and nitric oxide as alternative electron acceptors to oxygen, as well as the ability to use C_1_ compounds, such as methanol and methylamine, as electron donors to the respiratory chains. Different isolates of *P. denitrificans* have been described, but most of the molecular biology has been performed on a single strain, *P. denitrificans* PD1222 [12]. The soil denitrifier *P. denitrificans* PD1222 uses nitrate for several cellular processes, acting as: (i) a respiratory electron acceptor in anaerobic growth through the membrane-located nitrate reductase (Nar), the first enzyme of denitrification; (ii) an electron sink to dissipate reducing power under aerobic conditions via the periplasmic nitrate reductase (Nap); and (iii) a nitrogen source assimilated through the assimilatory nitrate reductase (Nas). The *P. denitrificans* PD1222 Nas system is encoded by the *nasABGHC* gene cluster, which is regulated in response to nitrate by a two-component system encoded by the *nasTS* genes located directly upstream from the nitrate transporter *nasA* gene [3,13]. NasS is a nitrate/nitrite sensor, and NasT presents an ANTAR domain, acting as a transcriptional anti-terminator in response to nitrate/nitrite [14,15]. In *P. denitrificans* PD1222, the role of the NtrBC system in regulating nitrate assimilation and polyhydroxybutyrate production has been demonstrated [16]. However, a contribution of the NtrYX system controlling nitrate assimilation could not be fully demonstrated [3]. The *P. denitrificans* NtrY protein is much larger than its counterpart, NtrB, suggesting that they may play different physiological roles [3]. 

*P. denitrificans* is a model organism for the characterization of the molecular control of the denitrification process. This bacterium carries out a complete denitrification pathway under oxygen-limited conditions through four consecutive reactions, whereby 10 electrons are consumed [17,18]. Recently, a holistic view of the denitrification process in *P. denitrificans* PD1222 has been provided by applying a proteomic gel-free approach (LC-MS/MS), using cells grown under anaerobic conditions with nitrate as the sole nitrogen and energy sources. This study allowed for the identification and quantification of the most represented peptides belonging to all the enzymes involved in denitrification. Additionally, the effects of environmental parameters, such as pH, on the proteome of *P. denitrificans* have been analyzed [19]. 

Iron is a relevant element in biological processes, and iron-containing proteins play important roles in oxidative and nitrosative stress protection, nitrogen metabolism, photosynthesis, methanogenesis, among others [20]. Both heme and non-heme iron-containing proteins are relevant for denitrification [21,22], including the respiratory nitrate reductase subunits NarG (4Fe-4S), NarH (4Fe-4S/3Fe-4S), and NarI (heme *b*), nitric oxide reductase subunits NorC (heme *c*) and NorB (heme *b*, heme *b*_3_, and Fe_B_), and nitrite reductase NirS (heme *cd*_1_). In many bacteria, including γ- and β-proteobacteria, bacilli, and cyanobacteria, the cellular iron homeostasis is mediated by the transcriptional regulator Fur (ferric uptake regulator), which represses hundreds of genes under iron-rich conditions. Additionally, several genes encoding iron-sulfur proteins are positively regulated by Fur through the repression of a small antisense RNA [23]. In *Bradyrhizobium diazoefficiens*, the global iron response regulator, Irr, which belongs to the Fur family, represses heme biosynthesis genes, Fe-S cluster biogenesis, and ferric siderophores uptake. In several rhizobial species, such as *Rhizobium* and *Sinorhizobium*, a Fur homolog that plays a role in repression of manganese uptake in response to elevated manganese concentrations has also been described [23]. Additionally, in these rhizobial species, a new global iron responsive regulator, named RirA, has been described. RirA belongs to the BadM/Rrf2 family of transcription factors, such as the *Escherichia coli* IscR protein that controls Fe-S cluster biogenesis and the NsrR proteins of γ- and β-proteobacteria. Under iron-rich conditions, RirA represses the expression of genes involved in ferrous iron and heme transport, siderophore biosynthesis and transport, and the synthesis of Fe-S centers [23]. 

In this work, the connection between iron availability and the functionality of electron transport components acting during denitrification has been investigated. For this purpose, a NtrY mutant of *P. denitrificans* PD1222 has been characterized through quantitative proteomics (LC-MS/MS), intracellular iron status, siderophore production, gene expression, and denitrification enzyme activities. The function of the two-component regulatory NtrYX system has been elucidated as a global iron-responsive regulator that controls both cellular iron homeostasis and energetic electron flux during denitrification.

## 2. Results

### 2.1. Physiological Characterization of the P. denitrificans NtrY Mutant under Denitrifying Conditions

**Figure 1:**
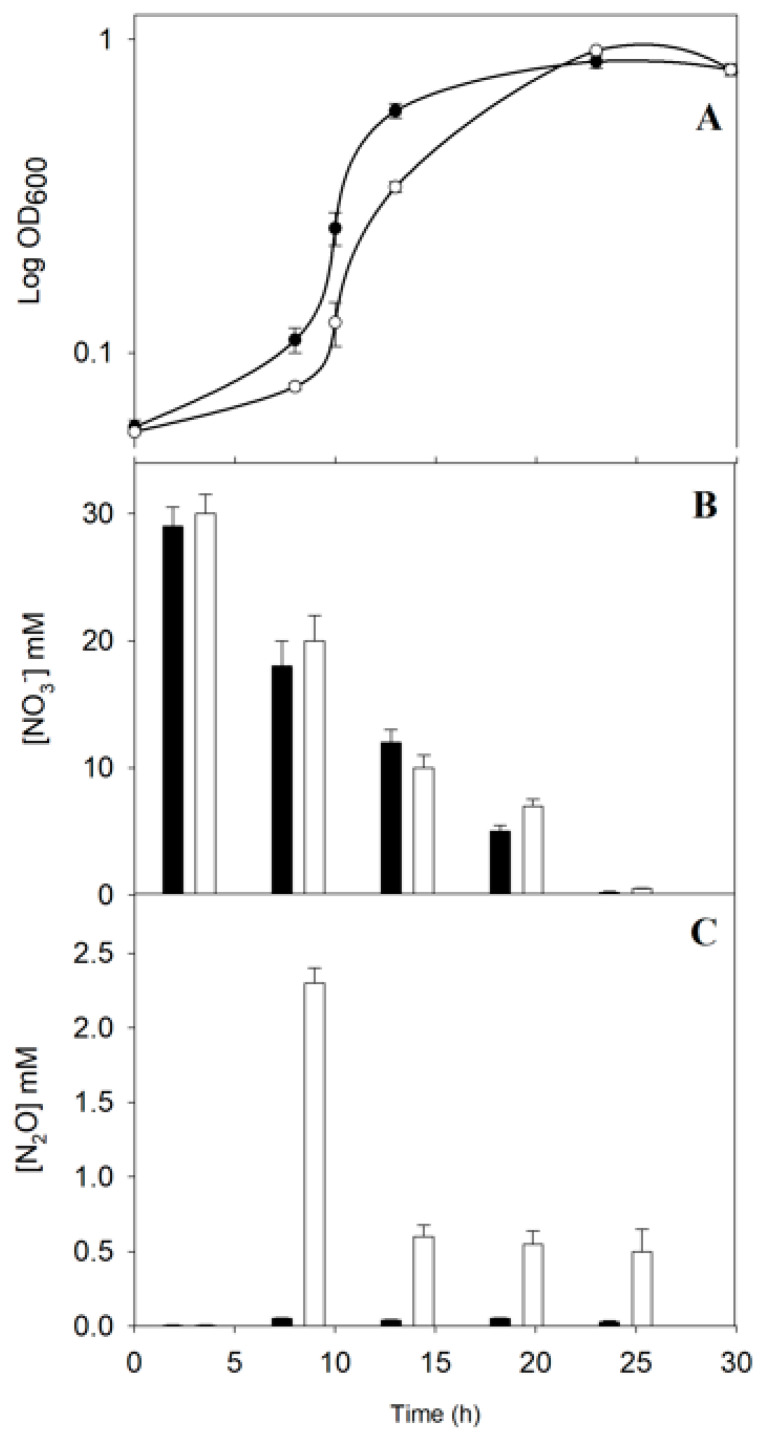
Physiological characterization of the *P. denitrificans* NtrY mutant under denitrifying conditions. The *P. denitrificans* wild-type strain (black) and NtrY mutant (white) were grown in minimal media with 30 mM nitrate as the sole nitrogen and energy source under anaerobic conditions. (**A**) Growth was determined by measuring the optical density at 600 nm. (**B**) Nitrate concentration in the media and (**C**) nitrous oxide production were determined as described in Materials and Methods section. Error bars correspond to data from three independent replicates (n = 3).

**Figure 2:**
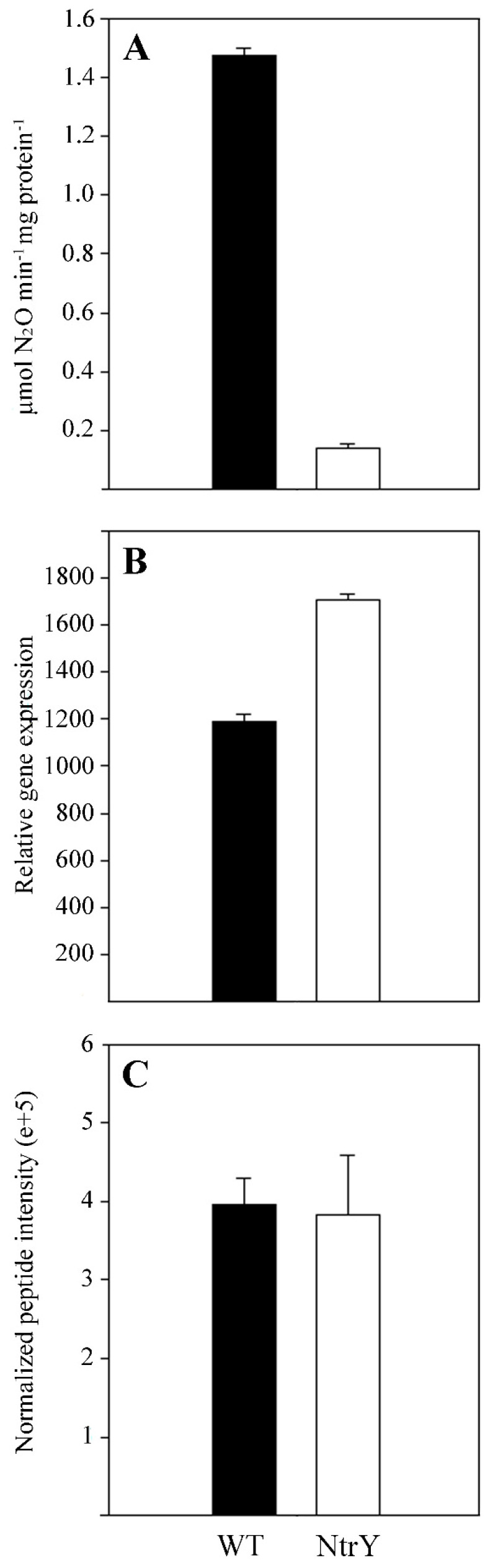
Nitrous oxide reductase in the *P. denitrificans* wild-type strain and NtrY mutant**.** The *P. denitrificans* wild-type strain (black) and NtrY mutant (white) were grown in minimal media with 30 mM nitrate as the sole nitrogen and energy source under anaerobic conditions. Cells were harvested upon reaching an OD_600_ of 0.3. (**A**) Nitrous oxide reductase activity was assayed as described in Material and Methods section. (**B**) Transcriptional expression of the *nosZ* gene was determined by qRT-PCR. (**C**) NosZ peptides quantification was obtained from the quantitative proteomic analysis. Error bars correspond to data from three independent replicates (n = 3).

In *P. denitrificans*, the *ntrBC* genes are clustered together with the *ntrYX* (Figure S1). A *P. denitrificans* mutant strain defective in the *ntrY* gene was generated in a previous work [3]. To study the effect of this mutation on the denitrification process, the wild-type strain and NtrY mutant were cultured in mineral salt media with 30 mM nitrate, as both the nitrogen source and final electron acceptor, under anaerobic denitrifying conditions. The *P. denitrificans* NtrY mutant showed a longer lag phase than the wild-type, although both strains displayed a similar final optical density (Figure 1A). Nitrate consumption, nitrite in the media, and nitrous oxide production were determined along the growth curves. Both the wild-type and NtrY mutant consumed nitrate (Figure 1B) and transiently accumulated nitrite in the media at similar rates. However, the NtrY mutant emitted elevated concentrations nitrous oxide, mainly at the early stage of the growth curve, while this gas was not detected in the wild-type strain (Figure 1C). The enzymic activities associated with respiratory (Nar), periplasmic (Nap), and assimilatory (Nas) nitrate reductases were determined in the wild-type strain and NtrY mutant. Significant differences were not found between the wild-type and NtrY mutant. However, when the nitrous oxide reductase activity (NosZ) was determined, it was found that this activity was reduced by about 10-fold in the NtrY mutant of *P. denitrificans* (Figure 2A). A quantitative gene expression analysis, using mRNA from wild-type and NtrY cells grown under denitrifying conditions, was carried out on the *narG*, *napA*, *nasC*, and *nosZ* genes, which code for the catalytic subunits of three type of nitrate reductases and nitrous oxide reductase. No significant differences in the gene expression levels were found between wild-type strain and NtrY mutant grown under denitrifying conditions (Figure 2B).

### 2.2. Quantitative Proteomic Analysis of the P. denitrificans NtrY Mutant under Denitrifying Conditions

To investigate the global changes on the proteome of *P. denitrificans* PD1222 derived from the mutation on the regulatory *ntrY* gene, a quantitative proteomic analysis by liquid chromatography–mass spectrometry/mass spectrometry (LC-MS/MS) has been performed from wild-type and NtrY mutant cells grown under anaerobic denitrifying conditions with 30 mM nitrate as both the nitrogen source and final electron acceptor. Cells were harvested when the cultures reached an optical density (OD) at 600 nm of about 0.3. Principal component analysis (PCA), clustering of the three biological replicates, and volcano plot are shown in the supplementary material (Figures S1–S3). In total, 2725 different proteins were identified in the wild-type strain, and 2843 proteins were identified in the NtrY mutant. From these, 16 proteins were ‘exclusive’ to the wild-type strain, whereas 134 proteins were ‘exclusive’ of the NtrY mutant, and 2709 proteins were shared between the wild-type and NtrY strains. The term ‘exclusive’ is used in this work to describe unique proteins found either in the wild-type strain or NtrY mutant. The overall number of identified proteins across both strains was 2859 from ~5100 putative structural genes present in the *P. denitrificans* genome (~56% of the total predicted gene products). A quantitative differential analysis was performed by comparison of the proteome from the wild-type strain to the NtrY mutant. In this comparative analysis, the protein profile obtained in the wild-type strain was considered the reference proteome, and the fold change (FC) was calculated as the ratio of wild-type/NtrY peptide intensities (Table S1). Proteins shared by both strains, wild-type and NtrY, but nevertheless differentially represented, were considered ‘over-represented’ in the wild-type strain if FC ≥ 2 or ‘down-represented’ in the wild-type strain if FC ≤ 0.5. In this differential analysis, 7 proteins were ‘exclusive’ of the wild-type strain, 24 proteins were ‘over-represented’ in the wild-type strain, 29 proteins were ‘down-represented’ in the wild-type strain, and 100 proteins were ‘exclusive’ of the NtrY mutant (Table S1). 

**Figure 3:**
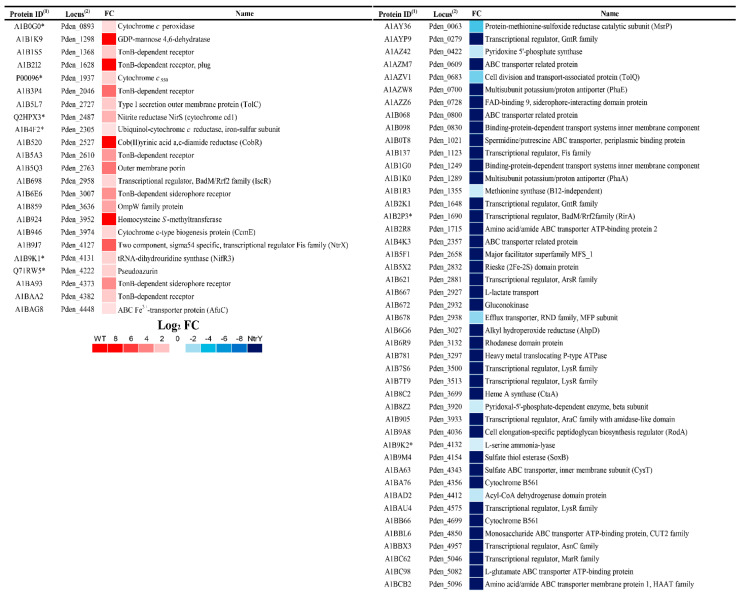
Heatmap of the comparative proteomic analysis of the wild-type strain versus the NtrY mutant of *P. denitrificans*. Differential proteomic analysis by LC–MS/MS of *P. denitrificans* wild-type strain and NtrY mutant was performed. Cells were grown anaerobically under denitrifying conditions, with nitrate as the sole nitrogen and energy source. Heatmap shows the fold changes (FC), represented as log_2_ normalized expression using the wild-type proteome as reference. After the *t*-test analysis was applied, the differential expressed proteins showed a *p*-value ≤ 0.05 and either a fold change ≥ 2 (exclusive or over-represented in the wild-type strain) represented in red or a fold change ≤ 0.5 (exclusive or over-represented in the NtrY mutant) represented in blue. ^(1)^ Protein code according to Uniprot database under the accession number UP000000361. ^(2)^ Genes annotated from GeneBank (T00440). (*) Detected only in two or one biological samples or displayed a high *p*-value.

Significant differences were not found in the nitrous oxide reductase NosZ peptide intensities when comparing wild-type strain to NtrY mutant (Figure 2C). A high number of proteins ‘exclusive’ or ‘over-represented’ in the wild-type strain of *P. denitrificans* were transporters, specifically the TonB-dependent transporters that correspond to the *P. denitrificans* (protein ID/gene *locus*) A1B2I2/Pden_1628, A1B3P4/Pden_2046, A1BA93/Pden_4373, A1B6E6/Pden_3007, A1B5A3/Pden_2610, A1BAA2/Pden_4382, and A1B1S5/Pden_1368 (Figure 3, Table S1). Other proteins involved in the transport of molecules across the membrane that were ‘over-represented’ in the wild-type strain were the membrane-bound component involved in multidrug efflux transport TolC (A1B5L7/Pden_2727) and outer-membrane protein OmpW (A1B859/Pden_3636). Several enzymes related to different metabolic processes were also ‘exclusive’ or ‘over-represented’ in the wild-type strain, such as the *cd*_1_-type nitrite reductase NirS (Q2HPX3/Pden_2487), cob(II)yrinic acid a,c-diamide reductase CobR (A1B520/Pden_2527) involved in the synthesis of cobalamin, homocysteine *S*-methyltransferase (A1B924/Pden_3952) that catalyses the reversible conversion of *S*-methylmethionine and L-homocysteine into two molecules of L-methionine, cytochrome *c*-type biogenesis protein CcmE (A1B946/Pden_3974) involved in cytochrome *c* maturation and its exportation to the periplasm, tRNA-dihydrouridine synthase NifR3 (A1B9K1/Pden_4131), and GDP-mannose 4,6-dehydratase (A1B1K9/Pden_1298). Additionally, the electron transport proteins cytochrome *c* peroxidase (A1BOGO/Pden_0893), cytochrome *c*_550_ (POOO96/Pden_1937), and pseudoazurin (Q71RW5/Pden_4222) were ‘over-represented’ in the wild-type strain. Interestingly, the iron responsive transcriptional regulator IscR (A1B698/Pden_2958) and σ^54^-specific transcriptional regulator NtrX (A1B9J7/Pden_4127) were also ‘over-represented’ in the wild-type strain (Figure 3, Table S1). 

In the NtrY mutant of *P. denitrificans* PD1222, ‘exclusive’ or ‘over-represented’ proteins also included a large number of transporters, such as amino acid/amide ABC transporter ATP-binding proteins (A1B2R8/Pden_1715 and A1BCB2/Pden_5096), sulfate ABC transporter inner membrane CysT (A1BA63/Pden_4343), siderophore-interacting FAD-binding protein (A1AZZ6/Pden_0728), monosaccharide ABC transporter ATP-binding protein (A1BBL6/Pden_4850), and L-glutamate transporter ATP-binding protein (A1BC98/Pden_5082). Enzymes, such as vitamin B_12_-independent methionine synthase (A1B1R3/Pden_1355), pyridoxine 5′-phosphate synthase (A1AZ42/Pden_0422), sulfate thiol esterase SoxB (A1B9M4/Pden_4154), alkyl hydroperoxide reductase AhpD (A1B6G6/Pden_3027), methionine-sulfoxide reductase MsrP (A1AY36/Pden_0063), 5-aminolevulinate synthase Heme A (A1B8C2/Pden_3699), and the cell division and transport-associated proteins TolR (A1AZV2/Pden_0684) and TolQ (A1AZV1/Pden_0683), were also ‘exclusive’ or ‘over-represented’ in the NtrY mutant. Additionally, two cytochromes *b*_561_ (A1BA76/Pden_4356 and A1BB66/Pden_4699) were ‘exclusive’ to the NtrY mutant (Figure 3, Table S1). Several regulatory proteins were also ‘exclusive’ to the NtrY mutant, such as three LysR-type proteins (A1BAU4/Pden_4575, A1B7S6/Pden_3500 and A1B7T9/Pden_3513), two GntR-type transcriptional regulators (A1B2K1/Pden_1648 and A1AYP9/Pden_0279), ArsR (A1B621/Pden_2881), AraC (A1B905/Pden_3933), Fis-like (A1B137/Pden_1123), AsnC (A1BBX3/Pden_4957), MarR (A1BC62/Pden_5046), and the iron-responsive repressor RirA (A1B2P3/Pden_1690). 

### 2.3. Intracellular Iron Content, Siderophore Production, and Gene Expression Analysis of P. denitrificans Iron-Responsive Regulators

The intracellular content of copper, cobalt, and iron was determined from wild-type and NtrY cells grown in mineral salt media under denitrifying conditions at the early exponential growth phase (OD_600_~0.3). No significant differences in the intracellular content of copper and cobalt were found when comparing the wild-type strain to the NtrY mutant. However, the intracellular content of iron was about 40% lower in the NtrY mutant (240 ± 59 μg Fe/kg dry cell weight) than in the wild-type strain (404 ± 51 μg Fe/kg dry cell weight).

**Figure 4:**
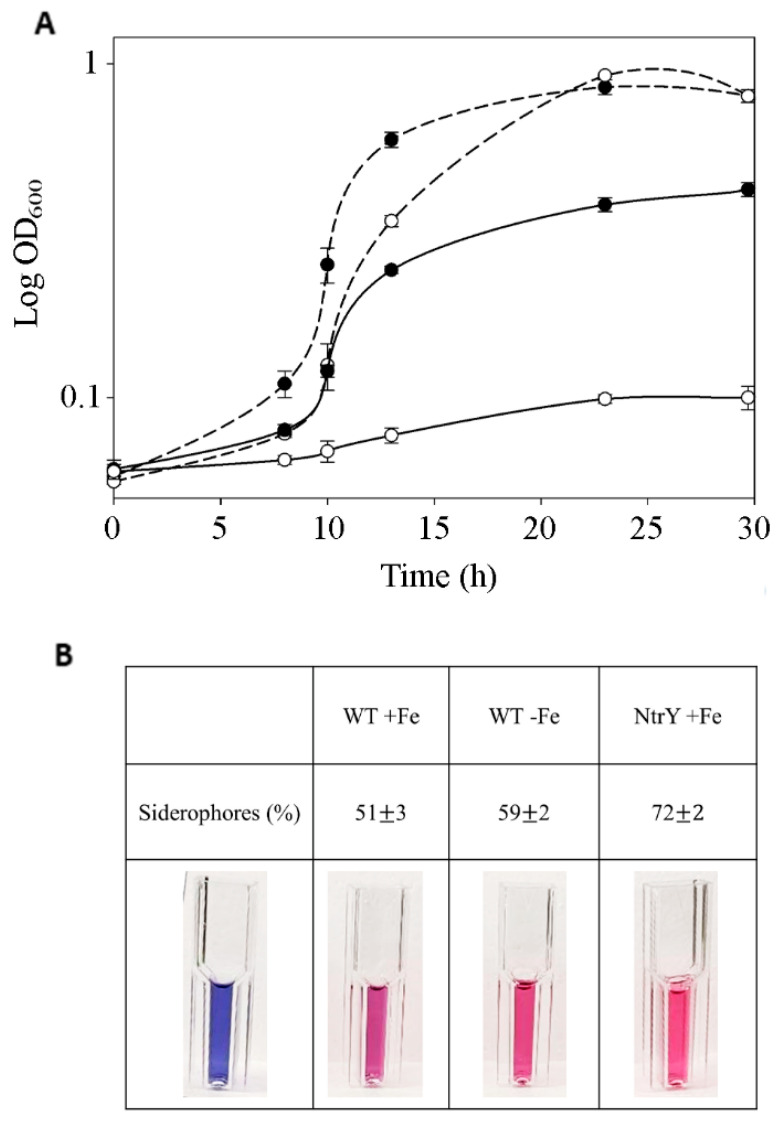
Growth and siderophores production of the *P. denitrificans* wild-type strain and NtrY mutant in iron-rich or iron-depleted medium. (**A**) The wild-type strain (black circles) and NtrY mutant (white circles) were grown under iron-rich conditions (discontinuous lines) or under iron-depleted conditions (continuous lines) in minimal media with 30 mM nitrate as the sole nitrogen and energy sources under anaerobic and denitrifying conditions. (**B**) Aliquots from the cultures (OD_600_~0.6) were taken and centrifuged, the resulting supernatants were used to assay siderophores production by using Chrome Azurol S (CAS), as described in Materials and Methods section. A control without addition of supernatant was performed (blue), the presence of siderophores was determined colorimetrically at 630 nm (pink). Error bars correspond to data from three independent replicates (n = 3).

To investigate a possible role of the *P. denitrificans* NtrYX system in controlling intracellular iron homeostasis, the wild-type strain, and the NtrY mutant were grown anaerobically under denitrifying conditions, either in the standard iron-rich minimal media or an iron-limited minimal media (without FeSO_4_ in the trace element solution). Under iron-rich conditions, the NtrY mutant showed a delayed growth, when compared to the wild-type strain, although it reached a similar final OD_600_ to the wild-type strain at the end of the exponential growth phase, as previously described. However, the NtrY mutant displayed very poor growth under iron-limited conditions (Figure 4A). Siderophore production was also determined in the extracellular media along the growth curves of the wild-type and NtrY mutant strains. This analysis revealed that, at the middle of the exponential growth (OD_600_~0.6), the wild-type strain produced more siderophores under iron-limited conditions than in the iron-rich media. When compared both strains, the NtrY mutant produced more siderophores than the wild-type strain (Figure 4B).

The genome of *P. denitrificans* PD1222 contains several putative iron-responsive regulatory genes, including Pden_1260 and Pden_4139 genes, which have been annotated as a manganese uptake regulator and as a ferric uptake regulator, respectively. These two genes belong to the Fur family and code for A1B1H1 and A1B9K9 proteins, respectively. A1B1H1 is homologous, displaying 42–44% identity to the iron responsive regulator Irr of *Rhizobium leguminosarum* bv. viciae and *Rhizobium etli* CFN 42, manganese responsive regulator Mur of *Rhizobium tropici* CIAT 899, and ferric uptake regulator Fur of *Bradyrhizobium* *diazoefficiens* (Table S2). A1B9K9 displayed no significant homology with iron-responsive regulators of α-proteobacteria deposited on the databases available. The A1B1H1 and A1B9K9 proteins shared about 30% identity (Table S2). The genome of *P. denitrificans* also contains three proteins, A1B2P3 (Pden_1690), A1B6G3 (Pden_3024), and A1B9P2 (Pden_4172), which are homologous, displaying 39–42% identity, to the iron-responsive repressor RirA of *Rhizobium etli*, *Sinorhizobium meliloti* SM11, *Brucella melitensis,* and *Agrobacterium tumefaciens* (reclassified as *Rhizobium radiobacter*) (Table S2). A1B2P3 (Pden_1690 gene) displayed about 47% identity to A1B6G3 (Pden_3024) and 45% identity to A1B9P2 (Pden_4172). In the quantitative proteomic analysis performed in this work, from the two RirA homologs found in the *P. denitrificans* genome, only the RirA protein encoded by the Pden_1690 gene was found to be ‘exclusive’ in the proteome of the NtrY mutant, as previously mentioned (Figure 3, Table S1). Additionally, a homolog to the iron-responsive regulator IscR of α-proteobacteria was found in the genome of *P. denitrificans*, A1B698 (Pden_2958), which showed the highest identity (about 47%) to the *Caulobacter vibrioides* CB15 IscR protein (Table S2).

A quantitative gene expression analysis of the *ntrYX* genes and other predicted iron-responsive regulator genes of *P. denitrificans* was carried out using mRNA isolated from the wild-type and NtrY mutant strains grown anaerobically under denitrifying conditions (Table 1). This transcriptional analysis by qRT-PCR revealed that expression of the *P. denitrificans ntrYX* genes was induced under iron-depleted conditions in the wild-type strain. Only one of the two *fur* homologs, Pden_4139, was induced by iron starvation, and this condition also caused the induction of the *rirA* and *iscR* genes in the wild-type strain of *P. denitrificans* (Table 1). In the NtrY mutant, which only grew under iron-rich conditions, expression of the *ntrX*, *fur*, *rirA*, and *iscR* genes was increased, compared to the wild-type strain grown in the presence of high iron concentration in the media (Table 1). The predicted Fur binding boxes were found in the promoter regions of the *ntrX*, *rirA, mur/irr*, and *iscR* genes (Table 1). The Fur recognition sequence detected, 5′-TG(C/A)-N-A-N8-CA(A/T)-3′, has been previously described for α-proteobacteria [23]. 

### 2.4. Identification of NtrX Binding Boxes and Target Genes in the Genome of P. denitrificans

A bioinformatic analysis was carried out in genome of the *P. denitrificans* PD1222 to identify predicted NtrX binding boxes in DNA regions located upstream from the predicted start codon, accordingly to the NtrX binding sequence 5′-C(A/T)-N_10_-GC-3′ previously described [10]. This analysis revealed that most of the proteins found in the quantitative proteomic study over- or down-represented in the NtrY mutant are encoded by genes that included a putative NtrX binding sequence upstream from their start codons (Table S1). Curiously, the *ntrY* gene also contains a putative Fur binding sequence, as mentioned above (Table 1). Additionally, putative NtrX binding sites were also found in the promoter regions of genes related specifically to denitrification, and, in general, the electron transfer flux that operates under denitrifying conditions (Figure S1). Expression of some of these genes was quantified by qRT-PCR in the wild-type strain and NtrY mutant of *P. denitrifcans* (Table 2). In the wild-type strain, the expression level of different genes targeted by NtrX was upregulated, when comparing iron-rich and iron-depleted conditions. These genes were the nitrate/nitrite-responsive transcriptional regulator *nasT* (Pden_4455), nitrate/nitrite transporter *narK* (Pden_4237), the nitrate transporter *nasA* (Pden_4453), small subunit of the nitric oxide reductase *norC* (Pden_ Pden_2484), and putative heme-containing *nosC* (Pden_4221), among others. Similarly, these genes were also upregulated in the NtrY mutant (Table 2). However, other genes that were also putative targets of NtrX changed their expression level only in the NtrY mutant of *P. denitrifcans* (Table 2). These genes were the transcriptional regulators *fnrP* (Pden_1850), *narR* (Pden_4238), and *nnrS* (Pden_4044), *cd*_1_-type nitrite reductase *nirS* (Pden_2487), cytochrome oxidase *cbb*_3_-type subunit I (Pden_1848), cytochrome *ba*_3_ quinol oxidase subunit 2 (Pden_5108), cytochrome *c*_550_ (Pden_1937), pseudoazurin (Pden_4222), cytochrome *c* peroxidase *ccP* (Pden_ Pden_0893), and acetyl-CoA synthetase (Pden_4550), among others (Table 2).

### 2.5. Phylogenetic Tree of the NtrYX System

**Figure 5:**
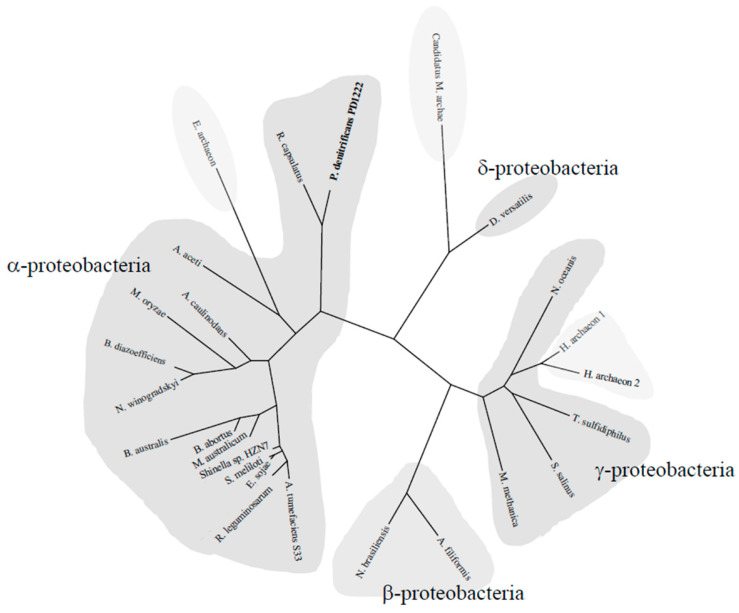
Phylogenetic tree of the NtrYX system in proteobacteria and archaea. The tree was constructed using the Phylogeny.fr platform [24]. Sequences were aligned with MUSCLE v3.8.31 with default settings. Ambiguous regions were removed with Gblocks (v0.91b). The phylogenetic tree was reconstructed using the maximum likelihood method implemented in the PhyML program (3.1/3.0 aLRT). Graphical representation and edition of the phylogenetic tree were performed with TreeDyn (v198.3). The *P. denitrificans* NtrY sequence is highlighted in bold. Protein sequences correspond to the following organisms and accession numbers: *Paracoccus denitrificans* PD1222 (A1B9J8), *Acetobacter aceti* (A0A1U9KGH8), *Agrobacterium tumefaciens* S33 (AMD59098), *Bartonella australis* (M1NY36), *Bradyrhizobium diazoefficiens* USDA 110 (Q89LQ4), *Brucella abortus* S19 (A0A0F6AQY7), *Rhizobium leguminosarum* bv. trifolii CB782 (AHG45292), *Mesorhizobium australicum* (L0KR80), *Methylobacterium oryzae* (A0A089Q4X3), *Sinorhizobium meliloti* 1021 (Q92Q88), *Ensifer sojae* (A0A249PA03), *Shinella* sp. HZN7 (A0A1A9G8A8), *Nitrobacter winogradskyi* (Q3SSN4), *Azorhizobium caulinodans* (Q04850), *Rhodobacter capsulatus* (D5AUA5), *Neisseria brasiliensis* (A0A5Q3RYY2), *Alysiella filiformis* (QMT30538), *Methylomonas methanica* (G0A7N2), *Nitrosococcus oceanis* (Q3J6T6), *Thioalkalivibrio sulfidiphilus* (B8GU15), *Spiribacter salinus* (R4VCU0), *Desulfuromonas versatilis* (BCR04177), *Candidatus Methanoperedenaceae archaeon* GB50 (CAD7776953.1), *Euryarchaeota archaeon* (MBM56026.1), *Halobacteria archaeon* (NNG12573.1), and *Halobacteria archaeon* (NNJ94214.1).

A bioinformatic analysis carried out to identify *P. denitrificans ntrY* gene (Pden_4128) orthologs in KEGG database revealed the presence of 917 homologs, which displayed the same *ntrYX* gene arrangement, and 674 of these *ntrY* gene-containing microorganisms (about 74%) presented the *ntrBC* genes in the same *locus*. According to this analysis, the NtrYX system is distributed mainly in proteobacteria (93%), with the phylum α-proteobacteria being the most abundant (74%) (Figure 5). The NtrYX system has been also found in other phyla, such as *Spirochaetes* (mainly chemoheterotrophic and anaerobic), *Acidobacteria*, *Deferribacteres* (anaerobes and ferric iron-reducers), and *Nitrospirae* (chemolithoautotrophic and nitrite-oxidizing bacteria). Furthermore, *ntrYX* homologs were also identified in archaea like *Euryarcheota archaeon*, *Candidatus Methanoperedenaceae archaeon* GB50, and *Halobacteria archaeon*, which were close homologs to those found in the phyla γ- and δ-proteobacteria (Figure 5). Additionally, about 40% of the microorganisms containing the *nrtYX* genes (366 from the 917 homologs) presented at least one of the four systems that are part of the denitrification process in their genome (Nar, Nir, Nor, or Nos). Of these, only 45 microorganisms (12.3%) were complete denitrifiers.

## 3. Discussion

### 3.1. Physiological Characterization of the P. denitrificans NtrY Mutant under Denitrifying Conditions

*Paracoccus denitrificans* PD1222 grows anaerobically under denitrifying conditions, with nitrate as the sole nitrogen source and electron acceptor [22,25]. Under these conditions, nitrate is not only reduced through the denitrification pathway to obtain energy, but it is also reduced via the assimilatory nitrate reductase (NasC) and nitrite reductase (NasBG) to produce ammonium, which is incorporated to carbon skeletons through the glutamine synthetase/glutamate synthase pathway [3,26]. In Gram-negative bacteria, *nas* genes are subjected to dual control by ammonia repression through the general nitrogen regulatory (NtrBC) system and specific nitrate/nitrite induction, which is mediated by different type of transcriptional regulators, depending on the microorganism [27]. In *P. denitrificans*, the NasS/NasT regulatory system operates in such a way that NasS acts as a nitrate/nitrite sensor and NasT as a transcriptional antiterminator that is active in the presence of these two N-oxyanions [14,27]. The NtrBC system plays different functions, including the control of glutamine synthetase, assimilatory nitrate and nitrite reductases, and nitrogenase [3,5]. In the genome of *P. denitrificans*, the *ntrYX* genes are located upstream from the *ntrBC* genes (Figure S1). The NtrYX proteins are homologous to NtrBC, and the interaction between the NtrBC and NtrYX systems has been described in *Azospirillum brasilense*, *Rhodobacter capsulatus*, and *Herbaspirillum seropedicae* [1,28,29,30,31,32]. Recently, a wide mutational analysis has been performed on the NtrYX system of different bacteria, including *P. denitrificans* PD1222 [3]. From this study, different functions for the NtrYX system have been proposed, such as controlling many different cellular processes, including nitrogen metabolism, respiration, biofilm formation, and osmotic pressure. However, the phenotype of these NtrY mutants remain unclear. To elucidate the function of the two-component system NtrYX in *P. denitrificans* PD1222, a previously constructed NtrY mutant strain [3] has been physiologically characterised in media containing nitrate as the sole nitrogen and energy source under denitrifying conditions (Figure 1). The NtrY mutant was able to grow with nitrate, which was consumed in a similar manner to the wild-type strain (Figure 1A,B). A significant concentration of nitrous oxide was emitted by the NtrY mutant, while this gas could not be detected in the wild-type strain (Figure 1C). The absence of nitrous oxide production in cultures of *P. denitrificans* wild-type strain has been previously reported, indicating that nitric oxide reductase (NorBC) and nitrous oxide reductase (NosZ) are very well matched, thus favouring the production of molecular nitrogen [33]. The emission of nitrous oxide by the NtrY mutant is consistent with the very low nitrous oxide reductase (NosZ) activity detected in this mutant (Figure 2A), highlighting that the mutation of the *ntrY* gene impairs complete denitrification. The expression of the *nosZ* gene determined by qRT-PCR and intensity of the NosZ peptides quantified in the proteomic analysis (Figure 2B,C), suggests that the mutation of the *ntrY* gene does not affect the transcription or translation of the *nosZ* gene, and low nitrous oxide reductase activity detected in the NtrY mutant could be related to post-translational regulation or a consequence of an indirect mechanism, such as a reduced electron supply to the nitrous oxide enzyme, as will be discussed below.

### 3.2. Proteomic Analysis, Iron Content and Siderophore Production of the P. denitrificans NtrY Mutant

To further characterise the NtrY mutant of *P. denitrificans*, a differential proteomic analysis has been carried out in the NtrY mutant, compared to the wild-type strain grown under denitrifying conditions. Cob(II)yrinic acid a,c-diamide reductase CobR was over-represented in the proteome of the wild-type strain, while vitamin B_12_-independent methionine synthase was over-represented in the NtrY mutant (Table S1). However, intracellular cobalt concentration determination revealed no significant differences between the wild-type and NtrY mutants, thus indicating that concentration of cobalamin in both strains could be similar. Another protein found deregulated in the proteomic study was the GDP-mannose 4,6-dehydratase, which was overproduced by the wild-type strain, when compared to the NtrY mutant (Table S1). This enzyme catalyses the first step in the conversion of GDP-mannose to GDP-fucose, which acts as a precursor of surface antigens in bacteria, such as the extracellular polysaccharide colanic acid in *Escherichia coli* [34]. By contrast, other proteins related to cell division, such as TolR and TolQ, were over-represented in the NtrY mutant (Table S1). The NtrYX system of the photosynthetic bacterium *R. sphaeroides* has been postulated to participate in controlling the transcription of the genes involved in cell envelope assembly, structure, and function, such as those specifically involved in peptidoglycan and extracellular polysaccharide synthesis, biosynthesis of O antigen and lipid A, putative lipoproteins, and cell division proteins, among others [9]. The bacterial cell envelope provides many important functions, including the protection of cells from harsh environments, acting as a selective permeability barrier, and playing a crucial role in biofilm formation, symbiosis, and virulence [9]. In the pathogen *Neisseria gonorreae*, the mutation in the *ntrX* gene negatively affects biofilm formation and virulence; it also causes a reduction in the expression of several genes involved in the electron transport chain, such as nitrite reductase, nitric oxide reductase, cytochrome *c* oxidase, and cytochrome *c* peroxidase [8]. In *Brucella* sp., the mutation of the *nrtY*/*ntrX* genes slightly affects the virulence, but genes encoding enzymes that participate in denitrification are severely impaired [6,7]. A role in redox sensing has been postulated for the NtrYX system of *Brucella*. In this bacterium, the NtrY protein binds a heme group, and its iron is rapidly oxidized to the ferric form in the presence of oxygen [2]. The 5-aminolevulinate synthase HemA was over-represented in the NtrY mutant (Table S1). The 5-aminolevulinate synthase catalyses the first step in the biosynthesis of tetrapyrroles, such as heme and cobalamin [35]. This is compatible with the presence of a heme group in the NtrY protein that could be attached to the polypeptide chain.

From all the proteins over-represented in the NtrY mutant, it is worth nothing the elevated number of TonB-dependent transporters were over-represented in the wild-type strain (Figure 3, Table S1). TonB-dependent transporters are outer membrane proteins that mainly bind and transport ferric chelates known as siderophores. These compounds are either simple molecules, such as citrate and catecholates, or complex structures, such as proteins [36,37]. The enhanced production of TonB-dependent siderophores by the NtrY strain, together with its lower intracellular iron content, when compared to the wild-type strain, suggest a novel role of the NtrYX system in controlling cellular iron homeostasis in response to iron starvation. This function has been demonstrated by the incapability of the NtrY mutant to grow in iron-depleted media (Figure 4A). This hypothesis is also supported by the fact that the mutation on the *ntrY* gene also caused an overproduction of siderophores, as revealed by the Chrome azurol S (CAS) assay used specifically for siderophores detection (Figure 4B). *P. denitrificans* may excrete to the media a catecholamine siderophore, L-parabactin, which has been described to be imported by an outer membrane receptor inside the cells that is specific for the import of ferric-parabactin [38]. Furthermore, transcription of *ntrYX* genes was induced in the wild-type strain of *P. denitrificans* in response to iron limitation, suggesting that NtrY may sense iron deficiency; in turn, it could phosphorylate NtrX to activate its binding to the promoter region of its target genes.

The results obtained in this work demonstrated that, in *P. denitrificans*, the NtrYX system plays a role as an iron-responsive regulator in controlling iron homeostasis, among other functions that will be discussed below.

### 3.3. Iron-Responsive Regulators of P. denitrificans under Denitrifying Conditions

To investigate the presence of other iron-responsive regulators, in addition to the NtrYX system, a bioinformatic analysis was performed in the genome of *P. denitrificans* PD1222 and homologs to the different iron-responsive regulators described in α-proteobacteria were identified (Table S2). Transcriptional expression analysis revealed that gene coding for the putative ferric uptake regulator Fur was induced under iron-depleted conditions in the wild-type, suggesting that this regulator could control Fur-dependent genes that may function under iron-limited conditions. However, another Fur homolog encoded by the *fur*/*mur*/*irr* gene, which is annotated as a manganese uptake regulator, did not change its expression level when comparing iron-depleted to iron-rich conditions (Table 1), thus suggesting that it could respond to manganese, rather than to iron, as has been previously described for α-proteobacteria that contains alternative iron-responsive regulators, such as RirA and Irr.

Evolution of iron and manganese regulons probably occurred in the common ancestor of the *Rhizobiales* and *Rhodobacterales*, where the Fur protein switched to regulating manganese transporters (Fur becomes Mur). In this sense, the two transcriptional regulators of *P. denitrificans* that belong to the BadM/Rrf2 family, RirA and IscR, were also upregulated under iron-depleted conditions (Table 1). The *fur*, *rirA*, and *iscR* genes were also upregulated in the NtrY mutant of *P. denitrificans,* but under iron-rich conditions (Table 1). This deregulation may suggest a role of NtrYX in controlling the expression of the other iron-responsive regulators in *P. denitrificans*. Additionally, a putative Fur binding sequence has been identified in the promoter region of the *P. denitrificans ntrY* gene, thus indicating a possible cross-talk between the NtrYX system and Fur in this bacterium. However, as previously described for NtrYX systems and Fur in other microorganisms, both transcriptional regulators could be acting as an inducer or a repressor of their targeted genes [9,23]. Furthermore, the iron-responsive regulators RirA and IscR seem to be under the control of the NtrYX system, as deduced by the putative NrtX binding box found in their promoter regions (Table 1). RirA and IscR contain an iron-sulfur cluster that could be unassembled under iron-limited conditions. RirA represses genes for ferrous iron and heme transport, siderophore biosynthesis, and the transport and synthesis of Fe-S centres. IscR has also been described to be involved in regulation of Fe-S biogenesis [23]. In addition to the NtrX binding sequences, putative Fur binding boxes could be found in the promoter regions of the genes encoding the iron-responsive regulators RirA and IscR (Table 1).

These results demonstrate a role of *P. denitrificans* NtrYX system in controlling the expression of the other iron-responsive regulators, including Fur, RirA, and IscR. Additionally, a cross-talk between the NtrYX system and Fur in this bacterium is proposed.

### 3.4. NtrX Targets Involved in Denitrification of P. denitrificans

Given that iron is an essential micronutrient that is very scarce in many environments for denitrifying microorganisms, the lack of studies on the impact of iron limitation on denitrification is surprising. Denitrifiers constitute a large population of microorganisms that are widely distributed among different ecosystems [39]. Several environmental parameters have been described to impact the optimal development of the denitrification process, such oxygen concentration, pH, and copper availability [22,25,40]. In particular, the relevance of copper-depleted soils has been directly related to nitrous oxide emissions, as consequence of an incomplete denitrification because the nitrous oxide reductase (NosZ) is a copper-containing enzyme [25]. In addition to the nitrous oxide reductase, the other three enzymes involved in denitrification (Nar, NirS, and Nor) are metalloenzymes that contain iron, either as iron-sulfur or heme centers. These iron-containing cofactors are essential for the functionality of each one of the four steps of this process. The integrated proteomic, transcriptional, and bioinformatic analyses performed in this work (Figure 3, Table 2 and Table S1) have demonstrated that the *cd*_1_-nitritrite reductase NirS, which reduces nitrite to nitrous oxide in the denitrification process, is upregulated by the NtrYX system in response to iron limitation. The induction of the *nirS* gene by the NtrYX system could be a mechanism to compensate for the disfunction of some iron-containing proteins involved in denitrification when iron is limited in the environment. In addition, two transcriptional regulators of the CRP/FNR family that control denitrification in response to different stimuli and contain an iron-sulfur cluster, FnrP and NarR, have been found upregulated in the NtrY mutant (Table 2). FnrP senses oxygen and nitric oxide, and this metallic center is also probably redox-sensitive [41,42,43]. A defective FnrP strain of *P. denitrificans* lacks respiratory nitrate reductase (Nar) and cytochrome *c* peroxidase (Ccp), and it shows also a decreased *cbb*_3_-type oxidase and increased *bb*_3_-type quinol oxidase [43]. NarR may respond to nitrate/nitrite, although the mechanism by which NarR regulates expression of *nar* genes remains unknown. Additionally, the heme/copper-containing NnrS-type transcriptional regulator family seems to be under the control of the NtrYX system in iron-depleted conditions (Table 2, Table S1). The *nnrS* gene is located on the *nar* gene *locus* (Figure S1). NnrS has been postulated as hypothetical nitric oxide sensor, but its role in controlling denitrification could not be demonstrated. However, a strong connection between the NnrS family proteins and nitric oxide, produced during denitrification or through other pathways, was supported from bioinformatic analyses [44]. A further study will be required to investigate the link between this type of transcriptional regulators in denitrification under iron-limited conditions.

The heme and non-heme centers of the FnrP, NarR, and NnrS transcriptional regulators could be damaged under iron-depleted conditions, and the NtrYX-mediated induction of these regulators could be considered a mechanism to balance the reduction of these transcriptional regulators at their native state of conformation. Additionally, in this integrated study, genes/proteins of the different iron-containing electron carrier proteins functioning during denitrification have been found induced in the wild-type strain, when compared to the NtrY mutant, including the cytochrome *bc*_1_ complex, cytochrome *cbb*_3_-type quinol oxidase, cytochrome *ba*_3_ quinol oxidase subunit 2, cytochrome *c*_550_, pseudoazurin, and cytochrome *c* peroxidase (Figure 3, Table 2 and Table S1). The direct control that the *P. denitrificans* NtrYX system seems to exert on the synthesis of electron transfer proteins that operate during denitrification could limit the electron flux that reaches the nitrous oxide reductase, thus causing nitrous oxide emission when the concentration of iron is not optimal iron in the environment (Figure 1 and Figure 2). Nitrous oxide emitted by the *P. denitrificans* NtrY mutant is maximum at an early state of the exponential phase (OD_600_~0.3), but the emission of this gas is partially reduced at later growth stages (Figure 1). This is probably related to the fact that the synthesis of some electron transfer proteins requires time to adapt to iron starvation, as well as the fact that, at a late exponential growth, the electron carrier proteins became adjusted to this condition, so that electron supply to the nitrous oxide reductase is partially recovered. Therefore, to ensure a complete denitrification process and, in turn, avoid nitrous oxide emissions, iron concentration in the environment should not be limited.

**Figure 6:**
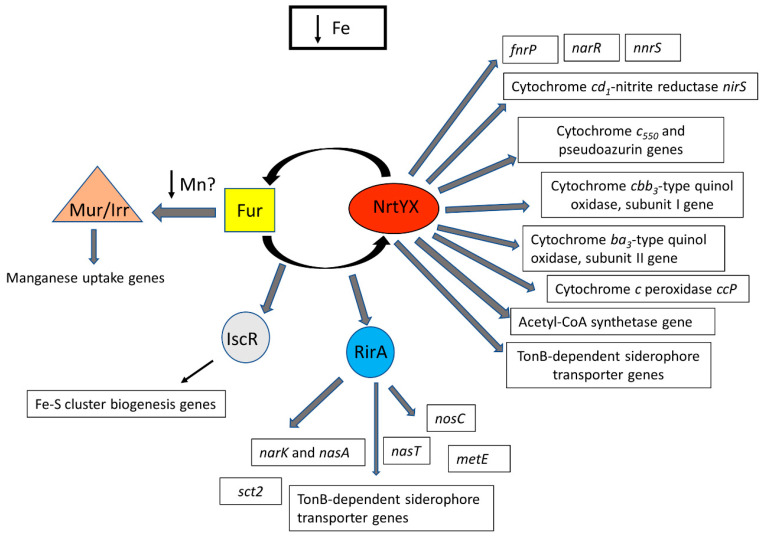
Hypothetical regulation network of the iron regulons in *P. denitrificans*. In this model, two major iron-responsive regulons have been included, NtrYX and Fur, which could display a cross-talk interaction. Other secondary transcriptional regulators that respond to iron limitation have also been regarded.

The integrated analysis carried out in this work has revealed that the *acs* gene that the codes for the acetyl-CoA synthetase show a putative NtrX-binding box in its promoter region and are induced in the NtrY mutant grown under denitrifying conditions (Table 2). The *acs* gene is clustered together with the nitrous oxide reductase and pseudoazurin genes (Figure S1). In a previous proteomic study, performed to elucidate the denitrification proteome of *P. denitrificans*, the acetyl-CoA synthetase was postulated as a key enzyme linking carbon/nitrogen metabolism during denitrification [19]. In this sense, the control of the *acs* gene by the NtrYX system (when the denitrification rate decreases), as a consequence of iron limitation, could be expected. In addition, there are a set of genes that are putatively targeted by NtrX that code for iron-containing proteins, including the small subunit of the nitric oxide reductase NorC, the protein NosC, which have been found upregulated both in the wild-type strain under iron-limiting conditions and NtrY mutant under iron-rich conditions (Table 2). The NtrYX system may also participate in controlling, under iron-limited conditions, two nitrate transporter genes of *P. denitrificans*, the nitrate/nitrite transporter *narK* gene for respiratory purposes, and nitrate transporter *nasA* gene for the assimilatory pathway (Table 2, Figure S1). This could be a mechanism to guarantee the maximal concentration of the specific N-oxyanion in the adequate subcellular compartment when it is required. In the regulation of these components, which also participate in the denitrification process, several iron-responsive regulators, including the NtrYX system, could be involved. The recruitment of two iron-responsive regulons, Fur and NtrYX, in *P. denitrificans* and biological relevance of their possible interaction is currently unknown (Figure 6). The *ntrYX* genes of *P. denitrificans* overlap by 3 bp and are co-transcribed, as described in other proteobacteria, such as *B. diazoefficiens* [3,9].

The results presented in this work reveal that the main targets of the *P. denitrificans* NtrYX regulon that are involved in denitrification include the iron-containing proteins nitrite reductase (NirS), two transcriptional regulators of the CRP/FNR family (FnrP and NarR), and several electron carrier proteins, such as cytochrome *bc*_1_ complex, cytochrome *cbb*_3_-type quinol oxidase, cytochrome *ba*_3_ quinol oxidase, cytochrome *c*_550_, pseudoazurin, and cytochrome *c* peroxidase.

### 3.5. Phylogenetic Distribution of the NtrYX Regulon

The phylogenetic distribution analysis of the NtrYX regulon reveals that this system is widely distributed among proteobacteria, mainly in α-proteobacteria. However, the NtrYX system is also generally found in other eubacteria and some archaea belonging to the Euryarchaeota phylum. Curiously, the NtrYX homologs found in these members of archaea display a high identity to those present in γ- and δ-proteobacteria (Figure 5), suggesting that these archaea could have acquired the NtrYX two-component regulatory system through a possible horizontal gene transfer event, conferring an enhanced adaptability on denitrifiers for survival in challenging environments, such as with low iron availability. Why the *ntrYX* genes are clustered together with the *ntrBC* genes, which display a specifically different role in controlling nitrogen assimilation, remains unknown. Perhaps this fact could be explained as a mechanism of *ntrYX* gene maintenance, considering that nitrogen assimilation is an essential process for survival of bacteria such as *P. denitrificans*.

## 4. Materials and Methods

### 4.1. Bacterial Strains, Media, and Growth Conditions

The NtrY mutant strain of *P. denitrificans* PD1222 was generated as previously described [3]. The *P. denitrificans* PD1222 wild-type strain and NtrY mutant were grown at 30 °C in mineral salt medium, as previously described [45], with 30 mM sodium succinate as carbon source and 30 mM potassium nitrate as nitrogen source and electron acceptor. When required, 37 μM Fe(SO_4_) was removed from the trace element solution and added to the standard media, which was considered as iron-depleted media (−Fe). Anaerobic cultures were performed in 50 mL-screw cap tubes filled with 50 mL media. Aerobic cultures (25 mL) were performed, with potassium nitrate (10 mM) as the sole nitrogen source and sodium succinate (30 mM) as the carbon source, on a shaker at 225 rpm. An aerobic overnight culture, prepared from a frozen stock in mineral salt medium supplemented with 10 mM ammonium chloride, was centrifuged and used as inoculum. Cell growth was followed by determining the optical density of the cultures at 600 nm (OD_600_), and cells were harvested at the optical density specified in each experiment. Antibiotic supplements, spectinomycin for the wild-type and NtrY mutant, and kanamycin for the NtrY mutant were used at 25 μg/mL final concentration in the media. 

### 4.2. In Vivo Determination of Nitrous Oxide Production

*P. denitrificans* PD1222 wild-type and NtrY mutant strains were cultured in minimal iron-rich media with nitrate (30 mM), as nitrogen and energy sources, in 30 mL-sealed tubes with 15 mL of Ar-anaerobic atmosphere. After inoculation, tubes were placed onto ice and sparged with argon for 1 h, until molecular nitrogen was not detected by GC. Cells were incubated at 30 °C for bacterial growth. At the indicated times, samples taken from the headspace were analysed by gas chromatography to determine N_2_O, as previously described [46]. Nitrate in the media was determined by using a method based on the incubation with sulfamic and perchloric acids [47].

### 4.3. Determination of Intracellular Metals Concentration by ICP-MS

To determine the intracellular content of iron, cobalt, and copper, 100 mL cultures of the wild-type strain and NtrY mutant of *P. denitrificans* were grown in minimal iron-rich media with 30 mM nitrate as the sole energy and nitrogen source and harvested upon reaching an OD_600_ of 0.3. Cells were washed in 20 mL of a buffer solution with Tris–HCl (20 mM, pH 8.0) and EDTA (4 mM). After centrifugation, pellets were dried (80 °C, 96 h), weighted, and subjected to digestion with high-purity nitric acid. Metal measurements were carried out by inductively coupled plasma mass spectrometry (ICP-MS, PerkinElmer Nexion350X) at the Central Service for Research Support (SCAI), University of Córdoba. Six different biological samples were analysed for each condition.

### 4.4. Detection of Siderophore Production

To determine production of siderophores by the wild-type and the NtrY mutant of *P. denitrificans*, cells were grown under iron-rich or iron-depleted conditions. The bacterial production of siderophores in the extracellular media was determined colorimetrically at 630 nm, as previously described [48]. The ternary complex chrome azurol S/iron(III)/hexadecyltrimethylammonium bromide (CAS-Fe_3_^+^/HDTMA) was used as indicator, which absorbs at 630 nm. This dye decolorates in the presence of a siderophore molecule, which acts as a strong chelator, removing the iron from this dye.

### 4.5. In Vitro Assays of Denitrification Enzymes 

*P. denitrificans* wild-type strains and NtrY mutant were grown in iron-rich minimal media under denitrifying conditions, as previously described. Cells were harvested upon reaching an OD_600_ of 0.3. Cells were washed in 20 mL of a buffer solution with Tris–HCl (20 mM, pH 8.0). Subcellular fractions were isolated from 35 mL-anaerobic cultures, as previously described [19]; periplasmic nitrate reductase (Nap), respiratory nitrate reductase (Nar), and assimilatory nitrate reductase (Nas) activities were determined, as previously described [19]. After isolation, the periplasmic fraction was stored on ice until used to determine Nap activity. The cytoplasmic fraction was used to determine Nas activity, and the membrane fraction was homogenized in 50 mM Tris-HCl (pH 8.0) to assay Nar activity. Nap activity was determined following detection of nitrite produced during the enzymatic reaction, as previously described [49]. Nar activity was measured following a microtiter protocol, previously described, by measuring oxidation of methyl viologen [15,50]. Nas activity was assayed in cytoplasmic fractions by measuring nitrite production, as previously described [19,32].

The periplasmic fraction was also used to measure the nitrite reductase (NirS) activity by following the protocol previously described [19], with minor modifications. The assay contained (in 1 mL final volume): 500 μL of a mixture with 2 mM methyl-viologen, 100 μM KNO_2_, and 50 mM Tris-HCl (pH 7.5), and it was incubated at 30 °C with 400 μL of periplasmic fraction and 100 μL of a solution containing 46 mM sodium dithionite (prepared in 1 M Tris-HCl, pH 7.5). The 100 µL-aliquots were taken from the assay at the following times: 0, 5, 10, 15, and 30 min. Disappearance of nitrite was monitored spectrophotometrically at 540 nm. Samples either without dithionite or without periplasmic fraction were used as negative control.

Nitrous oxide reductase (NosZ) activity was measured in vivo, as previously described in *P. denitrificans* [19], and nitrous oxide production was determined by gas chromatography, as previously described [46].

Data corresponding to enzymic activities was collected from three separated independent cultures. Protein concentration was estimated, as previously described, in subcellular fractions [51] or whole cells by a modified method of the Lowry procedure [52].

### 4.6. Quantitative Proteomic Analysis by LC-MS/MS

*P. denitrificans* wild-type and NtrY strains were grown in iron-rich minimal media under denitrifying conditions per triplicate. When cells reached an OD_600_~0.3, they were washed twice with 1 mL Tris–HCl (20 mM, pH 8.0) and resuspended in 300 μL of lysis buffer (8 M urea, Tris-HCl 50 mM in pH 7.5, 4% CHAPS). Afterwards, samples were disrupted by sonication in a Bandelin Sonoplus H2070 equipment (8 pulses for 20 s, at 25 W). To eliminate cell debris, extracts were centrifuged 12,000 rpm for 10 min at 4 °C and supernatants were precipitated by using the 2D clean-up kit (Amershamm GE Healthcare, London, UK). Then, proteins were dissolved in acetone and centrifuged at 12,000 rpm for 10 min. The 100 μL of RapiGest SF Surfactant (Waters Technologies, Milford, MA, USA) were added, and protein concentration was measured by using Qubit protein assay kit (Thermo Fisher Scientific, Washington, DC, USA), following the instructions of the manufacturer. A total of 10 μL of the protein solution were digested by using the iST kit (PreOmics, Munich, Germany). Peptides were diluted using LC-MS water 0.1% (*v*/*v*) formic acid to achieve a concentration of 10 ng/μL. A pool of samples was created by mixing the same volume of each sample. Peptides (200 ng) were loaded onto Evotips (Evosep, Odense, Demark), and Pierce™ Hela Tryptic Digest Standard (Thermo Fisher Scientific, Washington, DC, USA) was also prepared and loaded onto Evotips for quality control and system equilibration. Purified tryptic digests were separated by using the predefined 60 SPD method (21-min gradient time, 200 ng peptides) on an Evosep One LC system (Evosep, Odense, Demark) [53]. A fused silica 10-μm ID emitter (Bruker Daltonics, Billerica, MA, USA) was placed inside a nanoelectrospray source (CaptiveSpray source, Bruker Daltonics, MA, USA). The emitter was connected to an 8 cm × 150 μm reverse phase column, packed with 1.5 μm C18 beads. The column was heated to 40 °C in an oven compartment. Mobile phases were water and acetonitrile, buffered with 0.1% formic acid (LC-MS grade, Fisher Scientific). Liquid chromatography was coupled online to a TIMS Q-TOF instrument (timsTOF Pro, Bruker Daltonics, MA, USA) with data dependent acquisition–parallel accumulation serial fragmentation (ddaPASEF) for HeLa Digest and pooled QC analysis and data independent acquisition–parallel accumulation serial fragmentation (diaPASEF) for sample analysis through a CaptiveSpray nano-electrospray ion source [54,55]. For both acquisition modes, the ion mobility dimension was calibrated with three Agilent ESI-L tuning mix ions (*m*/*z*, 1/K0: 622.0289 Th, 0.9848 Vs cm^−2^; 922.0097 Th, 1.1895 Vs cm^−2^; 1221.9906 Th, 1.3820 Vs cm^−2^). The collision energy decreased linearly from 59 eV at 1/K0 = 1.6 Vs cm^−2^ to 20 eV at 1/K0 = 0.6 Vs cm^−2^. To carry out the DDA-PASEF method, each top N acquisition cycle consisted of four PASEF MS/MS. The accumulation and ramp times were set to 100 ms. Singly charged precursors were excluded from fragmentation using a polygon filter in the (*m*/*z*, 1/K0) plane. All precursors that reached a target value of 20,000 were excluded for 0.4 min. Precursors were isolated using a Q window of 2 Th for *m*/*z* < 700 and 3 Th for *m*/*z* > 800. For DIA-PASEF, the ‘long gradient’ method (m/z range: 400–1200 Th, 1/K0 range: 0.6–1.6 Vs cm^−2^, DIA-PASEF windows: 16 × 25 Th) was applied. The experiment was analysed running first 10 QCs to condition the system and following the sequence HeLa–QC–10 samples analysis–HeLa–QC. The order of the samples was randomly defined. 

Data from the FASTA *Paracoccus denitrificans* PD1222 strain (Uniprot, UP000000361) was used to build an in-silico library with DIA-NN 1.8 (https://github.com/vdemichev/DiaNN/releases/tag/1.8, accessed on 8 October 2021). The options ‘FASTA digest for library-free search/library generation’, ‘Deep learning-based spectra’, and ‘RTs and IMs prediction’ were enabled. Missed cleavages were set to 1, precursor change range 2–4, and precursor m/z range 100–1700, neural network classifier set to double-pass mode, quantification strategy was set to ‘Any LC (high accuracy)’, and match between runs (MBR) option was also enabled. MS1 and MS2 accuracy and retention time window scans were set to 0 to let DIA-NN to perform its automatic inference for the first run in the experiment. Following previously published recommendations [56], DIA-NN output was filtered at precursor *q*-value < 1% and global protein *q*-value < 1%. FDR validation benchmark was filtered to include only unmodified peptides or peptides with carbamidomethylated cysteines, oxidated methionine, or excised N-terminal from methionine. The numbers of precursors/proteins were obtained based on filtering the library for precursors within charge range from 2 to 4 and mass range 100.0–1700.0 *m*/*z*. All other DIA-NN settings were left default, using RT-dependent cross-run normalization and filtering the output at 1% FDR. The number of threads used by DIA-NN was 52, as automatically suggested by the software. Finally, for DIA-PASEF analysis, spectral library generated in the previous step was added. MS1 and MS2 accuracy and retention time window scans were set to 0 to let DIA-NN to perform their automatic inference for the first run in the experiment. Protein inference in DIA-NN was configured to use the protein names from FASTA file (the same used for the generation of the spectral library) with enabled MBR. When reporting protein numbers and quantities, Protein.Group column in the DIA-NN report was used to identify the protein group, and PG-MaxLFQ was used to obtain the normalized quantity. Quantification mode was set to ‘Any LC (high accuracy)’. All other settings were set as described above for the generation of the spectral library.

Data analysis was performed by using Perseus software (1.6.12.1) (https://maxquant.org/perseus/, accessed on 11 October 2021). Firstly, an exploratory analysis was carried out, and a PCA analysis, heat-map, and volcano plot were generated (Figures S2–S4) by using default parameters. Then, a *t*-test was applied, and differential expressed proteins were defined as those with a *p*-value ≤ 0.05 and either a fold change ≥ 2 (exclusive or over-represented in the wild-type strain) or ≤0.5 (exclusive or over-represented in the NtrY mutant). Proteins indicated as exclusively expressed were identified in at least two of the three replicates of one condition and undetectable in the other condition. Once data were filtered, a GO enrichment analysis was carried out by using the ComparativeGO application [57]. Data were deposited to the ProteomeXchange Consortium (http://proteomecentral.proteomexchange.org; accessed on 12 May 2022) via the PRIDE partner repository with the dataset identifier PXD033855.

### 4.7. Gene Expression Analysis by qRT-PCR

Wild-type strain and NtrY mutant were grown in iron-rich or -limited minimal media under denitrifying conditions, as previously described. Cells were harvested and washed in TEG buffer containing 25 mM Tris-HCl (pH 8.0) with 1% glucose and 10 mM EDTA. RNA isolations were performed using the Direct-zol RNA Miniprep kit (Zymo Research, Irvine, CA, USA). DNase incubation was carried out in the column with RNase-free DNase (Zymo Research, Irvine, CA, USA), and an additional post-column treatment was required with DNase I (Ambion-Thermo Fisher Scientific, Waltham, MA, USA). The concentration and purity of the RNA samples were measured by using a ND1000 spectrophotometer (Nanodrop Technologies, Waltham, MA, USA). Synthesis of total cDNA and PCR reactions were carried out with specific primers (Table S3), as previously described [19]. Data were normalized with *dnaN* used as housekeeping gene, and the specific sequence primers were *dnaN*-F: 5′-CATGTCGTGGGTCAGCATAC-3′ and *dnaN*-R: 5′-CTCGCGACCATGCATATAGA-3′. The ∆∆Ct method has been used to calculate the relative fold gene expression.

### 4.8. Bioinfomatic Analisis of the NtrX Binding Sequence in the Genome of P. denitrificans

The pattern locator (https://www.cmbl.uga.edu/software/patloc.html; accessed on 11 January 2022), previously described [58], was used for finding NtrX sequence patterns in the genome of *P. denitrificans* PD1222, with the restriction of searching in “intergenic” regions. For that purpose, 5′-C(A/T)-N_10_-GC-3′ sequence was used as putative NtrX binding box, as previously described, in *R. sphaeroides* [10]. The NtrX binding box was searched in the genome of *P. denitrifcans* applying a r-scan statistics to outputs.

### 4.9. Statistical Analysis

Statistical significance was determined by a two-tailed *t*-test analysis, considering that pair of samples were different when the *p*-value was lower than 0.05. Perseus (v1.6.12.1) software was used for the proteomic data analysis. Other data were compared using the IBM SPSS Statistics v22 software (https://www.ibm.com/support/pages/spss-statistics-220-available-download; accessed on 1 July 2022).

## 5. Conclusions

The results presented in this work demonstrate that the two-component NtrYX system of *P. denitrificans* is an iron-responsive transcriptional regulator that constitutes a key regulatory element to optimize cellular iron homeostasis and different uses of nitrate (as nitrogen and energy source) under anaerobic and denitrifying conditions if iron concentration fluctuates in the environment, mainly when iron availability is very scarce. This role of the NtrYX system has been highlighted by the proteome, low cellular content of iron, and high nitrous oxide concentration emitted by the NtrY mutant of *P. denitrificans*. This role of the NtrYX system in controlling iron homeostasis and complete denitrification process in response to iron limitation could be also carried out by the NtrYX system present in other microorganisms.

## Figures and Tables

**Table 1:** Transcriptional analysis by qRT-PCR of putative iron-responsive regulators of *P. denitrificans*.

Locus ^1^	Protein ID ^2^	Name	NtrX Binding Sequence (Distance from Start Codon)	Fur Binding Sequence (Distance from Start Codon)	qRT-PCR		
					WT		NtrY
					+Fe	−Fe	+Fe
Pden_4128	A1B9J8	PAS/PAC sensor signal transduction histidine kinase (*nrtY*)	**CA**CAGAACGGCC**GC** (-33) **GT**GGCCGTTCAG**CG** (-44)	**TGA**C**A**CGGCGCCG**CAA** (-92)	0.3 ± 0.1	3.1 ± 0.1	-
Pden_4127	A1B9J7	Two-component, σ^54^-specific, transcriptional regulator, Fis family (*ntrX*)	-	-	1.1 ± 0.6	3.7 ± 0.9	2.3 ± 0.4
Pden_1260	A1B1H1	Manganese uptake regulator, Fur family (*mur/irr*)	**CT**TGTGCTCTGG**GC** (-82)	**TGA**G**A**TCGTCCGC**CA**C (-29)	0.1 ± 0.1	0.7 ± 0.2	1.0 ± 0.4
			**CT**TCGTTCTGCT**GC** (-69)				
			**CT**GCCGTTATCT**GC** (-59)				
			**CT**CAACTGTCAA**GC** (-37)				
			**CT**TCGCATCTGG**GC** (-11)				
Pden_4139	A1B9K9	Putative ferric uptake regulator, Fur family (*fur*)	**CT**TGGCGTTTCC**GC** (-53)	-	5.3 ± 0.4	28.0 ± 1.3	8.4 ± 0.3
Pden_1690	A1B2P3	Transcriptional regulator, BadM/Rrf2famil (*rirA*)	**CA**AGGTTGCGCG**GC** (-98)	**TGC**A**A**TCAGGATG**CAT** (-19)	1.8 ± 0.2	8.9 ± 0.7	12.7 ± 1.2
			**CT**GCAAGGTTGC**GC** (-101)	**TG**TATTCTGGATA**CAT** (-56)			
Pden_2958	A1B698	Transcriptional regulator, BadM/Rrf2family (*iscR*)	**CT**TGCCTATGTC**GC** (-78)	**TGC**CGCGGCCCCT**CA**C (-51)	1.0 ± 0.5	4.3 ± 0.7	2.8 ± 0.7
			**GA**TCTAGAGCCG**CG** (-111)				

^1^ Protein annotated from UniProt (UP000000361). ^2^ Genes annotated from GeneBank (T00440). Fur box in α-proteobacteria: 5′-TG(C/A)-N-A-N8- CA(A/T)-3′. NtrX binding box: 5′-CA(N_10_)GC-3′. Conserved nucleotides in NtrX and Fur boxes are highlighted in bold.

**Table 2:** Transcriptional analysis by qRT-PCR of putative NtrX target genes of *P. denitrificans*.

Locus ^1^	Protein ID ^2^	Name	NtrX Binding Sequences (Position from Start Codon)	qRT-PCR		
				WT		NtrY
				+Fe	−Fe	+Fe
Pden_0893	A1B0G0	Cytochrome *c* peroxidase (Ccp)	**GT**GGGCGCGTCT**CG** (-78) **GT**CAGATGTTTT**CG** (-133) **GT**TCTGCCTTGC**CG** (-149) **GA**CGGTCCGTCG**CG** (-108)	0.4 ± 0.2	1.1 ± 0.3	8.4 ± 0.9
Pden_1355	A1B1R3	Methionine synthase B12-independent (MetE)	**CA**TGCCACTGGC**GC** (-201) **CA**AGGTGACATC**GC** (-77) **CA**TCGCCGCTTC**GC** (-69) **CT**GTTTCCTCAG**GC** (-19) **CT**CAGAAGGCAT**GC** (-3) **GT**TTCCTCAGGC**CG** (-17) **GA**CCGCGGTGGG**CG** (-111) **GA**GCGGCCCATC**CG** (-91) **GA**TATGCAAGGACG (-7)	0.8 ± 0.4	4.3 ± 0.9	36.5 ± 2.3
Pden_1848	Q51679	Cytochrome *c* oxidase, *cbb*_3_-type, subunit I	**CT**TAAATCCTGC**GC** (-11) **GT**CACACGGTTT**CG** (-86) **GA**CTTTGATCTG**CG** (-103) **CA**ATCTGTCATT**GC** (-118) **CA**GGATGTCGCA**GC** (-163) **CA**GGTGAAACTT**GC** (-211)	0.6 ± 0.3	1.86 ± 0.3	7.5 ± 0.5
Pden_1850	A1B353	Putative transcriptional regulator, Crp/Fnr family (FnrP)	**CA**AGGTTCCAGC**GC** (-125) **CT**CATCGCCTTC**GC** (-18) **CT**GCTGCGTCGC**GC** (-78)	0.3 ± 0.1	1.1 ± 0.2	4.1 ± 0.9
Pden_1937	P00096	Cytochrome *c*_550_	**CA**CAATGATCTT**GC** (-61) **CA**TGATCCGCAG**GC** (-39)	0.4 ± 0.1	1.2 ± 0.1	6.9 ± 0.2
Pden_2046	A1B3P4	TonB-dependent receptor	**GA**TCCCTTGTCC**CG** (-105) **CT**CGCCCTCTCG**GC** (-37)	0.5 ± 0.1	13.2 ± 1.1	5.4 ± 0.1
Pden_2305	A1B4F2	Ubiquinol-cytochrome *c* reductase iron-sulfur subunit (cytochrome *bc*_1_)	**CT**GCGGCGATTT**GC** (-119) **GT**TCCGTCGTAT**CG** (-20) **GT**CGTATCGCCC**CG** (-15) **GA**TCGCTAGAAC**CG** (-64)	0.6 ± 0.2	1.1 ± 0.1	8.9 ± 0.6
Pden_2484	Q51662	Nitric oxide reductase subunit C (NorC)	**CA**AGCGTGAGTC**GC** (-47) **GA**CCTCACTGTC**CG** (-32)	0.2 ± 0.1	3.8 ± 0.3	12.5 ± 1.2
Pden_2486	O33432	Protein NirI	**GT**CAAAGCCCCG**CG** (-59) **GA**ACGGCGTGAA**CG** (-89)	3.2 ± 0.6	3.4 ± 0.1	3.4 ± 0.6
Pden_2487	Q2HPX3	Nitrite reductase NirS	**GT**CAAAGCCCCG**CG** (-59) **GA**ACGGCGTGAA**CG** (-89)	4.4 ± 0.9	5.0 ± 0.9	24.6 ± 2.2
Pden_2610	A1B5A3	TonB-dependent receptor	**GT**CGGCAGGCTG**CG** (-155)	0.2 ± 0.1	1.4 ± 0.4	5.0 ± 0.9
Pden_2832	A1B5X2	Rieske [2Fe-2S] domain protein (Stc2)	**CA**GCCGAATGTC**GC** (-176) **CT**GCCGTAACTT**GC** (-54) **CT**CCGTCCGGTC**GC** (-286)	0.3 ± 0.9	3.6 ± 0.2	5.2 ± 0.9
Pden_3027	A1B6G6	Alkylhydroperoxide reductase (AhpD)	**GA**CGCTTGCCGC**CG** (-58)	0.5 ± 0.1	1.7 ± 0.4	5.2 ± 0.4
Pden_4044	A1B9B6	NnrS family protein	**GA**GCCGGTGCCA**CG** (-30) **GA**GGGGCCGCAT**CG** (-10)	0.2 ± 0.1	0.3 ± 0.1	6.7 ± 0.7
Pden_4213	A1B9T3	Acetyl-coenzyme A synthetase (Acs)	**GT**ACGGGACATG**CG** (-221) **GA**AAACCGATTG**CG** (-75)	0.4 ± 0.1	0.6 ± 0.1	2.1 ± 0.7
Pden_4221	A1B9U1	NosC protein	**GT**TTATGGATCG**CG** (-74) **GT**CCCGACCCTG**CG** (-194) **GA**AGGAGAATCG**CG** (-35) **GA**GTTTTTTCCT**CG** (-231)	1.6 ± 0.8	3.1 ± 0.2	12.5 ± 0.9
Pden_4222	Q71RW5	Pseudoazurin	**GA**AGGAGAATCG**CG** (-287) **GA**GTTTTTTCCT**CG** (-91) **GT**TTATGGATCG**CG** (-248) **GT**CCCGACCCTG**CG** (-128)	0.5 ± 0.1	0.7 ± 0.1	20.0 ± 1.3
Pden_4237	A1B9V7	Nitrate/nitrite transporter NarK	**CT**CAAATCGTCA**GC** (-150) **GT**CCGGCCGGCC**CG** (-89) **GA**TTGGGACTTT**CG** (-12) **GA**CTTCTCAAAT**CG** (-145) **GA**TTTTTGCAAG**CG** (-221)	6.8 ± 0.8	8.8 ± 0.13	45.3 ± 3.5
Pden_4238	A1B9V8	Putative transcriptional regulator, Crp/Fnr family (NarR)	**CT**CAAATCGTCA**GC** (-150) **GT**CCGGCCGGCC**CG** (-89) **GA**TTGGGACTTT**CG** (-12) **GA**CTTCTCAAAT**CG** (-145) **GA**TTTTTGCAAG**CG** (-221)	0.4 ± 0.1	1.2 ± 0.4	6.1 ± 0.3
Pden_4453	A1BAH3	Major facilitator superfamily MFS_1, nitrate transporter NasA	**CT**GATGGCGAAG**GC** (-18) **CT**GTCGGAAAGC**GC** (-31) **CT**GGGTCAGGAC**GC** (-176) **CT**CCGCCCGAAA**GC** (-235) **GT**CGCGGGAATC**CG** (-104)	1.9 ± 0.2	53.4 ± 4.3	14.7 ± 0.1
Pden_4455	A1BAH5	Response regulator receiver ANTAR domain protein NasT	**GT**CCCGGTTTTG**CG** (-82) **GA**ACCCCGCGCC**CG** (-38)	1.0 ± 0.3	7.5 ± 1.6	2.9 ± 0.9
Pden_4719	A1BB86	Periplasmic nitrate reductase subunit (NapE)	**GA**GGCGTTTTGA**CG** (-16)	0.2 ± 0.1	2.2 ± 0.1	2.1 ± 0.3
Pden_5108	A1BCC4	Cytochrome *ba*_3_ quinoloxidase subunit 2	**CA**GGTGCGGACG**GC** (-25) **CA**AGCTCGCCAT**GC** (-103) **CT**GAGTCGCAGG**GC** (-38) **GA**TGTCGGTGAG**CG** (-10) **GA**GCGACAGGTG**CG** (-19) **GA**CAGGTGCGGA**CG** (-23) **GA**AACGCCGCGG**CG** (-154)	0.3 ± 0.1	1.1 ± 0.7	6.9 ± 0.16

^1^ Protein annotated from UniProt (UP000000361). ^2^ Genes annotated from GeneBank (T00440). Conserved nucleotides in NtrX and Fur boxes are highlighted in bold.

## Data Availability

Not applicable.

## Supplementary Materials

The following supporting information can be downloaded at: https://www.mdpi.com/article/10.3390/ijms23169172/s1.
