# Supplementary material for: The NtrYX Two-Component System of Paracoccus denitrificans Is Required for the Maintenance of Cellular Iron Homeostasis and for a Complete Denitrification under Iron-Limited Conditions
Source: Int J Mol Sci. 2022 Aug 15;23(16):9172. doi: 10.3390/ijms23169172 (PMC9409073; doi:10.3390/ijms23169172)
Supplement: Supplementary file 1 [file ijms-23-09172-s001.zip › ijms-1853499-supplementary.pdf]

Table S1. Differential quantitative proteomic analysis of the wild-type strain *versus* NtrY^{-} mutant of *P. denitrificans* PD1222 grown under denitrifying conditions. Location of putative NtrX binding boxes in gene promoter regions encoding affected proteins.

**Protein ID**[1]	**Locus**[2]	**Name**	**FC**[3]	***p*-value**	**NtrX binding sequence**	**Start position**	**End position**	**Orientation**[4]	**Distance from start codon**[5]
A1AZG8	Pden_0550	Lytic transglycosylase, catalytic	WT	-	**GT**GGCGAT CCGCC**CG**	523185	523198	C	-131
A1B1K9	Pden_1298	GDP-mannose 4,6-dehydratase	WT	-	**CA**CCATTT AACC**GC**	1261145	1261158	D	-13
					**CA**GCCGCA AGCG**GC**	1261201	1261214	D	-69
					**CA**TGCCGC CCGG**GC**	1261243	1261256	D	-111
					**CA**ATGGCG CCGC**GC**	1261268	1261281	D	-136
A1B2I2	Pden_1628	TonB-dependent receptor, plug	WT	-	**GA**GGGCTT CACG**CG**	1610353	1610366	C	-40
					**CT**GAACCG GGTA**GC**	1610382	1610395	D	-69
A1B338	Pden_1835	Uncharacterized protein	WT	-	**CA**CCCATC CCTT**GC**	1834875	1834888	D	-167
A1B520	Pden_2527	Cob(II)yrinic acid a,c-diamide reductase	WT	-	**CA**GCCGTG ACCC**GC**	2539924	2539937	D	Pden_2524
A1B924	Pden_3952	Homocysteine S-methyltransferase	WT	-	**GT**CCGCTC GCGG**CG**	1088315	1088328	C	-73
A1BBZ8	Pden_4982	Short-chain dehydrogenase/reductase	WT	-	Not detected	-	-	-	-
A1B3P4	Pden_2046	TonB-dependent receptor	25.26	2.70E-05	**GA**TCCCTT GTCC**CG**	2053803	2053816	C	-105
					**CT**CGCCCT CTCG**GC**	2053871	2053884	D	-37
A1B5Q3	Pden_2763	Outer membrane porin	23	4.40E-04	**CA**GGCAAG GATC**GC**	2783522	2783535	D	-221
A1BA93	Pden_4373	TonB-dependent siderophore receptor	13.2	7.20E-05	**GT**ATTTAT GGAA**CG**	1280822	1280835	C	Pden_4372
A1B9J7	Pden_4127	Two component, sigma54 specific, transcriptional regulator Fis family (NtrX)	11.8	5.92E-05	Not detected	-	-	-	-
A1B6E6	Pden_3007	TonB-dependent siderophore receptor	11.53	4.00E-05	**GA**ATGCTG GCTG**CG**	180571	180584	C	-34
					**GA**TTTGCG ATTC**CG**	180584	180597	C	-47
					**GA**AACCGC CGAG**CG**	180610	180623	C	-73
					**GA**GCGACA GCGC**CG**	180619	180632	C	-96
A1B5A3	Pden_2610	TonB-dependent receptor	10.38	1.70E-04	**GT**CGGCAG GCTG**CG**	2620681	2620694	C	-155
A1B859	Pden_3636	OmpW family protein	7.76	3.40E-04	**GA**AAGGAT GATG**CG**	787163	787176	C	-108
A1B2V1	Pden_1748	MltA-interacting MipA family protein	4.78	2.40E-04	**CA**GAACCC GCTT**GC**	1737216	1737229	D	-51
					**GA**AGGGG CGTTT**CG**	1737232	1737245	C	-35
					**GA**CGCAGG GTTC**CG**	1737245	1737258	C	-22
A1B571	Pden_2578	Uncharacterized protein	4.52	8.90E-04	Not detected	-	-	-	-
A1B5L7	Pden_2727	Type I secretion outer membrane protein, TolC family	3.47	5.30E-04	**CA**AGAACC TCCG**GC**	2745391	2745404	D	Pden_2726

A1BAA2	Pden_4382	TonB-dependent receptor	3.35	1.80E-03	**CT**TGGCCTTTCC**GC**	1575003	1575016	D	-51
					**GT**AGGCGGTGAG**CG**	1574827	1574840	C	-227
					**GT**GAGCGAAGGC**CG**	1574834	1574847	C	-220
					**GT**CGCCATCCCG**CG**	1574978	1574991	C	-76
					**GA**CGGGCAAGGC**CG**	1574796	1574809	C	-258
					**GA**AGGCCGAAGG**CG**	1574840	1574853	C	-214
					**GA**CCTGCCGCAG**CG**	1574908	1574921	C	-146
A1B1S5	Pden_1368	TonB-dependent receptor	3.35	2.30E-03	**CA**GAAATGCTCT**GC**	1337262	1337275	D	-41
					**CT**CTGCGGAAAA**GC**	1337270	1337283	D	-49
					**CT**GGCGCACCCC**GC**	1337338	1337351	D	-117
					**GA**ACAGGTCAGG**CG**	1337493	1337506	C	-275
Q9R9L0*	Pden_4222	Pseudoazurin	2.93	3.78E-03	**GA**AGGAGAATCG**CG**	1385239	1385252	C	-287
					**GA**GTTTTTTCCT**CG**	1385435	1385448	C	-91
					**GT**TTATGGATCG**CG**	1385278	1385291	C	-248
					**GT**CCCGACCCTG**CG**	1385398	1385411	C	-128
A1B9V1	Pden_4231	Uncharacterized protein	2.88	1.60E-03	Not detected	-	-	-	-
A1B043	Pden_0775	50S ribosomal protein L18	2.88	5.80E-04	**GA**TCGGCTTTAA**CG**	753186	753199	C	-36
A1B9K1	Pden_4131	tRNA-dihydrouridine synthase (NifR3)	2.87	2.35E-02	**GA**AAGAAAACGG**CG**	1288467	1288480	C	-32
					**GA**CGGAATTTGC**CG**	1288510	1288523	C	-75
					**GA**GCTTCAAGTC**CG**	1288580	1288593	C	-145
					**CT**TTCCGCGCCA**GC**	1288636	1288649	D	-201
P00096*	Pden_1937	Cytochrome c550	2.86	2.83E-03	**CA**CAATGATCTT**GC**	1935525	1935538	D	Pden_1936
					**CA**TGATCCGCAG**GC**	1935547	1935560	D	
A1B391	Pden_1890	Ribonuclease D	2.79	1.40E-04	**CT**TGTAACAGCA**GC**	1893373	1893386	D	Pden_1892
A1B946	Pden_3974	Cytochrome c-type biogenesis protein (CcmE)	2.61	2.60E-03	**CT**GCCGCAAGCC**GC**	1112756	1112769	D	Pden_3973
A1B698	Pden_2958	Transcriptional regulator, BadM/Rrf2 family (IscR)	2.59	7.30E-04	**CT**TGCCTATGTC**GC**	127693	127706	D	-64
					**GA**TCTAGAGCCG**CG**	127726	127739	C	-97
Q2HPX3*	Pden_2487	Nitrite reductase NirS (cytochrome cd1)	2.53	1.00E-02	**GT**CAAAGCCCCG**CG**	2503931	2503944	C	-54
					**GA**ACGGCGTGAA**CG**	2503961	2503974	C	-24
A1B0G0*	Pden_0893	Cytochrome c peroxidase	2.31	2.00E-02	**GT**GGGCGCGTCT**CG**	877144	877157	C	-78
					**GT**CAGATGTTTT**CG**	877199	877212	C	-133
					**GT**TCTGCCTTGC**CG**	877215	877228	C	-149
					**GA**CGGTCCGTCG**CG**	877174	877187	C	-108
A1B4F2*	Pden_2305	Ubiquinol-cytochrome c	2.10	3.62E-03	**CT**GCGGCGATTT**GC**	2303754	2303767	D	-119

		reductase iron-sulfur subunit							
					**GT**TCCGTC GTAT**CG**	2303853	2303866	C	-20
					**GT**CGTATC GCCC**CG**	2303858	2303871	C	-15
					**GA**TCGCTA GAAC**CG**	2303809	2303822	C	-64
A1BAG8	Pden_4448	ABC transporter related protein	2.09	6.30E-04	**CT**AAACTA ATTA**GC**	1656656	1656669	D	-44
A1B3Z2	Pden_2144	Thymidylate kinase	0.46	2.00E-03	**CA**GCGTTC ACTT**GC**	2143646	2143659	D	Pden_2143
A1B9D6	Pden_4064	Uncharacterized protein	0.45	1.80E-03	**CT**GCCTTC ATGC**GC**	1218576	1218589	D	-53
					**CT**TCATGC GCCC**GC**	1218580	1218593	D	-49
					**CT**GCAAAA CTCC**GC**	1218627	1218640	D	-2
A1B8Z2	Pden_3920	Pyridoxal-5'-phosphate-dependent enzyme, beta subunit	0.44	2.10E-03	**CT**TGTGGA CCAA**GC**	1057157	1057170	D	Pden_3921
A1B589	Pden_2596	Mutator MutT protein	0.44	5.70E-04	Not detected	-	-	-	-
A1B1R3	Pden_1355	Methionine synthase (B12-independent)	0.43	1.00E-03	**CA**TGCCAC TGGC**GC**	1319848	1319861	D	-201
					**CA**AGGTGA CATC**GC**	1319972	1319985	D	-77
					**CA**TCGCCG CTTC**GC**	1319980	1319993	D	-69
					**CT**GTTTCC TCAG**GC**	1320030	1320043	D	-19
					**CT**CAGAAG GCAT**GC**	1320046	1320059	D	-3
					**GT**TTCCTC AGGC**CG**	1320032	1320045	C	-17
					**GA**CCGCGG TGGG**CG**	1319938	1319951	C	-111
					**GA**GCGGCC CATC**CG**	1319958	1319971	C	-91
					**GA**TATGCA AGGA**CG**	1321054	1321067	C	-7
A1B3E3	Pden_1944	Peptidyl-dipeptidase Dcp, Metallo peptidase	0.41	2.00E-03	**CT**TTTCCG GTTG**GC**	1947257	1947270	D	-9
A1B3N2	Pden_2034	Toluene tolerance family protein	0.38	1.90E-03	Not detected	-	-	-	-
A1BAD2	Pden_4412	Acyl-CoA dehydrogenase domain protein	0.38	2.10E-03	**CT**TTCAAA CCGG**GC**	1613831	1613844	D	-31
A1B502	Pden_2509	RNA pyrophosphohydrolase	0.38	1.20E-03	Not detected	-	-	-	-
A1B0J8	Pden_0931	Extracellular solute-binding protein, family 5	0.36	1.70E-03	**CA**GATTAA CCGC**GC**	912848	912861	D	-100
					**CA**TTCGCG CCAT**GC**	913038	913051	D	-291
					**CT**CATGAC TAGC**GC**	912958	912971	D	-210
A1B8X3	Pden_3901	ATPase associated with various cellular activities, AAA_3	0.34	2.80E-04	Not detected	-	-	-	-
A1AZE1	Pden_0523	tRNA 2-selenouridine synthase	0.28	2.40E-05	**GT**TGATCC CGGG**CG**	493347	493360	C	Pden_0522
A1B4M0	Pden_2374	Two component transcriptional regulator, winged helix family	0.28	6.40E-05	**CA**GCGGTT TGCA**GC**	2373062	2373075	D	-54

					**CT**GTGGCG CGGC**GC**	2373038	2373051	D	-30
A1B430	Pden_2182	Uncharacterized protein	0.27	1.30E-03	Not detected	-	-	-	-
A1AZ44	Pden_0424	Tetratricopeptide TPR_2 repeat protein	0.27	2.70E-03	**GA**TGGCCG GGGG**CG**	398160	398173	C	Pden_0425
A1B249	Pden_1493	ABC transporter related protein	0.27	8.50E-04	**CA**GTAGCG GTAC**GC**	1457042	1457055	D	-40
					**GA**GTGAAT GAGT**CG**	1457020	1457033	C	-18
A1B1W4	Pden_1407	Uncharacterized protein	0.25	1.30E-04	**CT**TTACCC CGAC**GC**	1376453	1376466	D	-1
A1AZV2	Pden_0684	Cell division and transport-associated protein (TolR)	0.25	5.70E-04	**CT**GGGCGG TTCT**GC**	664787	664800	D	Pden_0683
A1B5G2	Pden_2669	Outer-membrane lipoprotein carrier protein	0.24	1.30E-03	**CA**CATCAC GTTC**GC**	2680111	2680124	D	-32
					**CT**CCAAAG ACAT**GC**	2680098	2680111	D	-19
					**GT**TCGCTG GATG**CG**	2680119	2680132	C	-40
A1B310	Pden_1807	Extracellular solute-binding protein, family 5	0.2	2.90E-04	**CA**TCCTTG CGCG**GC**	1802101	1802114	D	-157
A1B678	Pden_2938	Efflux transporter, RND family, MFP subunit	0.2	3.00E-04	**CT**TGCGGT CCCG**GC**	103918	103931	D	-191
					**GT**CCTGAT GGGA**CG**	103836	103849	C	-109
					**GT**CTTGCG GTCC**CG**	103916	103929	C	-189
A1B1T9	Pden_1382	Protease Do	0.19	2.90E-03	**CA**TGCCGG GGCG**GC**	1351508	1351521	D	-16
					**CA**AATGAA CCAC**GC**	1351577	1351590	D	-85
A1B5K4	Pden_2714	Peptidase M16 domain protein	0.17	2.20E-03	**GT**GATCGG CGCG**CG**	2732614	2732627	C	Pden_2715
A1AZX3	Pden_0705	Lytic transglycosylase, catalytic	0.17	1.00E-04	Not detected	-	-	-	-
A1BBB0	Pden_4743	Peptidoglycan-binding domain 1 protein	0.16	3.10E-03	**CA**CGTTCG CATC**GC**	227995	228008	D	-162
					**CA**CGCGCT CCTC**GC**	228153	228166	D	-4
					**GT**CGGCCC CGGC**CG**	228114	228127	C	-43
A1AZV1	Pden_0683	Cell division and transport-associated protein (TolQ)	0.15	3.50E-04	**CT**GGGCGG TTCT**GC**	664787	664800	D	-56
A1B003	Pden_0735	Carboxymethylen ebutenolidase	0.14	3.10E-03	**CA**GCAGGG CGGG**GC**	719259	719272	D	-27
A1B5K5	Pden_2715	Peptidase M16 domain protein	0.11	3.00E-04	**GT**GATCGG CGCG**CG**	2732614	2732627	C	-80
A1AY36	Pden_0063	Protein-methionine-sulfoxide reductase catalytic subunit (MsrP)	0.09	1.70E-03	**CA**TCGGCA TCCG**GC**	56371	56384	D	-43
					**GA**AGCGA AGGGG**CG**	56298	56311	C	-116
A1AYH7	Pden_0207	Alpha/beta hydrolase fold protein	NtrY	-	**CA**GAGGCG AAAC**GC**	191151	191164	D	-26
					**GA**AGGGC AGAGG**CG**	191145	191158	C	-20
A1AZW8	Pden_0700	Multisubunit potassium/proton antiporter, (PhaE)	NtrY	-	**CA**TCGCCT AGTT**GC**	677798	677811	D	Pden_0697

A1AZZ6	Pden_0728	FAD-binding 9, siderophore-interacting domain protein	NtrY	-	**CA**AGGTCA ACGG**GC**	712033	712046	D	-47
A1B127	Pden_1113	Amidase, hydantoinase/carb amoylase family	NtrY	-	**CA**CGCGGA AGCC**GC**	1090914	1090927	D	-27
					**CT**TGTCCG GCAC**GC**	1090905	1090918	D	-18
					**CT**CCTCCT TCCC**GC**	1091052	1091065	D	-19
					**CT**CCTTCC CGCC**GC**	1091055	1091068	D	-168
					**GA**CCGCAA CTCC**CG**	1091022	1091035	C	-136
					**GA**CCGGCA GATC**CG**	1091124	1091137	C	-237
A1B2K1	Pden_1648	Transcriptional regulator, GntR family	NtrY	-	Not detected	-	-	-	-
					Not detected	-	-	-	-
A1B2R8	Pden_1715	Amino acid/amide ABC transporter ATP-binding protein 2, HAAT family	NtrY	-	**GT**TACCGT CAGG**CG**	1704106	1704119	C	Pden_1717
A1B3I4	Pden_1986	Cardiolipin synthetase 2	NtrY	-	**CA**ATTCGG CACC**GC**	1990646	1990659	D	-56
					**CT**GCGTGG AATT**GC**	1990633	1990646	D	-43
A1B3W6	Pden_2118	Uncharacterized protein	NtrY	-	Not detected	-	-	-	-
A1B416	Pden_2168	Putative arginyl-tRNA-protein transferase	NtrY	-	**CA**GCCGAA ACGC**GC**	2173379	2173392	D	-83
					**CT**CAGCCC CAGA**GC**	2173345	2173358	D	-103
					**GT**CAATGC CGCG**CG**	2173266	2173279	C	-183
					**GA**TGGACC GGAA**CG**	2173280	2173293	C	-168
					**GA**ATGACC CGAC**CG**	2173401	2173414	C	-47
A1B4V2	Pden_2459	OmpA/MotB domain protein	NtrY	-	Not detected	-	-	-	-
A1B5F5	Pden_2662	Conserved hypothetical integral membrane protein	NtrY	-	Not detected	-	-	-	-
A1B621	Pden_2881	Transcriptional regulator, ArsR family	NtrY	-	**GT**TATCGG TTGG**CG**	45372	45385	C	-159
					**GT**GATAGG GACT**CG**	45388	45401	C	-143
					**GT**TAAGCA AAGC**CG**	45531	45544	C	-1
					**GA**TAGGGA CTCG**CG**	45390	45403	C	-141
A1B667	Pden_2927	L-lactate transport	NtrY	-	**GT**TCCGCC GTTG**CG**	90553	90566	C	-25
					**GT**CCTGCG CGGT**CG**	90575	90588	C	-47
A1B672	Pden_2932	Gluconokinase	NtrY	-	Not detected	-	-	-	-
A1B6D1	Pden_2992	Uncharacterized protein	NtrY	-	**CT**CCTGTT TTCC**GC**	166493	166506	D	-8
					**GT**ATGGAA CAAC**CG**	166575	166588	C	-90
					**GA**ACAACC GCCG**CG**	166580	166593	C	-95
A1B7U3	Pden_3517	Urocanate hydratase	NtrY	-	Not detected	-	-	-	-
A1B839	Pden_3616	Uncharacterized protein	NtrY	-	Not detected	-	-	-	-

A1B905	Pden_3933	Transcriptional regulator, AraC family with amidase-like domain	NtrY	-	**CT**GGAATC CGCG**GC**	1070748	1070761	D	-13
					**CT**TTTCCT TTGG**GC**	1070793	1070806	D	-58
					**GT**CTGGAA TCCG**CG**	1070746	1070759	C	-11
A1B988	Pden_4016	Uncharacterized protein	NtrY	-	**CT**GAACCC GACG**GC**	1163975	1163988	D	Pden_4017
A1B9A8	Pden_4036	Cell elongation-specific peptidoglycan biosynthesis regulator (RodA)	NtrY	-	Not detected	-	-	-	-
A1B9M4	Pden_4154	Sulfate thiol esterase (SoxB)	NtrY	-	**CT**GCGGCC GCCG**GC**	1309602	1309615	D	-100
					**CT**TGCCCG AAAC**GC**	1309684	1309697	D	-18
					**GA**CCCCAA CCTT**CG**	1309630	1309643	C	-72
					**GA**CGCGCC TTGG**CG**	1309658	1309671	C	-44
A1BA39	Pden_4319	Uncharacterized protein	NtrY	-	**CT**GAGGCC GCCG**GC**	1500280	1500293	D	Pden_4321
A1BA63	Pden_4343	Sulfate ABC transporter, inner membrane subunit (CysT)	NtrY	-	**GA**CAGAGT TTAT**CG**	1528009	1528022	C	Pden_4344
A1BA76	Pden_4356	Cytochrome B561	NtrY	-	Not detected	-	-	-	-
A1BA91	Pden_4371	Uncharacterized protein	NtrY	-	**GA**GCCTCC CTGT**CG**	1562578	1562591	C	-41
A1BAB0	Pden_4390	Carbohydrate ABC transporter membrane protein 1, CUT1 family	NtrY	-	**GT**TCGCGC CTGT**CG**	1588987	1589000	C	Pden_4393
A1BAS3	Pden_4554	Uncharacterized protein	NtrY	-	**CT**GCGGGG CGCG**GC**	35592	35605	D	Pden_4553
A1BAU4	Pden_4575	Transcriptional regulator, LysR family	NtrY	-	Not detected	-	-	-	-
A1BBC5	Pden_4758	Branched-chain alpha-keto acid dehydrogenase E1 component	NtrY	-	**GA**GATTTT GCGG**CG**	252159	252172	C	-
A1BBL6	Pden_4850	Monosaccharide ABC transporter ATP-binding protein, CUT2 family	NtrY	-	**GA**CGCCCT GCGG**CG**	351591	351604	C	-39
A1BBY2	Pden_4966	Uncharacterized protein	NtrY	-	**CA**GGCGGC ATCT**GC**	474009	474022	D	Pden_4965
A1BC46	Pden_5030	Uncharacterized protein	NtrY	-	**CA**ATGGAA GCAG**GC**	542789	542802	D	-36
					**CA**TCCTTC GCCG**GC**	542898	542911	D	-145
					**CT**CCAAGG GAGC**GC**	542819	542832	D	-66
					**GA**CCGGCA CCAG**CG**	543030	543043	C	-277
A1BC98	Pden_5082	L-glutamate ABC transporter ATP-binding protein	NtrY	-	**GT**GTCCCG GCCG**CG**	595041	595054	C	-22
A1BCB2	Pden_5096	Amino acid/amide ABC transporter membrane protein 1, HAAT family	NtrY	-	**GA**CCGGGC CCGA**CG**	608824	608837	C	-40
A1AYW5	Pden_0345	Uncharacterized protein	NtrY	-	**CT**CTCTCG TTAC**GC**	328044	328057	D	-194
					**GA**TTGCTG AATT**CG**	327993	328006	C	-245

A1B4K3	Pden_2357	ABC transporter related protein	NtrY	-	Not detected	-	-	-	-
A1AYG6	Pden_0194	Uncharacterized protein	NtrY	-	**GA**TGGCCG ATCT**CG**	176639	176652	C	-29
					**CA**GGTGTT TATG**GC**	176789	176802	D	-164
					**CT**GCTTGG TCCC**GC**	176734	176747	D	-109
					**GT**CCCGCC GGTT**CG**	176741	176754	C	-116
A1AYP9	Pden_0279	Transcriptional regulator, GntR family	NtrY	-	**CA**GGCCGT TTTG**GC**	257567	257580	D	-125
					**CA**TAGCAT TATG**GC**	257661	257674	D	-30
					**CT**CGTCGG CTTG**GC**	257587	257600	D	-104
A1AZ69	Pden_0449	Exonuclease, RNase T and DNA polymerase III	NtrY	-	Not detected	-	-	-	-
A1AZD2	Pden_0514	Sarcosine oxidase, delta subunit, heterotetrameric	NtrY	-	**CA**TCCGGG CCCG**GC**	484077	484090	D	-205
					**CA**AGCCGC CCCC**GC**	484128	484141	D	-154
					**CA**GCGGCC TTGC**GC**	484157	484170	D	-125
					**CA**TCGACC TTCT**GC**	484220	484233	D	-62
A1AZL8	Pden_0600	Putative glutamine amidotransferase	NtrY	-	**CT**TTTCGT GCTA**GC**	576716	576729	D	-16
A1AZM7	Pden_0609	ABC transporter related protein	NtrY	-	**CT**GAATGC CGCA**GC**	584692	584705	D	-32
					**GA**AACAA GTTTC**CG**	584639	584652	C	-71
A1B068	Pden_0800	ABC transporter related protein	NtrY	-	Not detected	-	-	-	-
A1B098	Pden_0830	Binding-protein-dependent transport systems inner membrane component	NtrY	-	**CT**AGTCTC GCGC**GC**	909565	909578	D	Pden_0829
A1B0B5	Pden_0847	Polysaccharide biosynthesis protein	NtrY	-	Not detected	-	-	-	-
A1B0I8	Pden_0921	NAD kinase	NtrY	-	**CT**GGCCAT GATG**GC**	902907	902920	D	-3
					**GA**CTGCGC GCGG**CG**	903708	903721	C	-18
A1B0T8	Pden_1021	Spermidine/putrescine ABC transporter	NtrY	-	**CA**CGATCT CCTA**GC**	1000137	1000150	D	-76
					**GA**TTGGAA GATC**CG**	1000108	1000121	C	-106
A1B137	Pden_1123	Transcriptional regulator, Fis family	NtrY	-	Not detected	-	-	-	-
A1B159	Pden_1145	Intracellular protease, PfpI family	NtrY	-	**CA**AAAGG GCAGG**GC**	1122202	1122215	D	-40
					**GA**CGGACC TGTC**CG**	1122178	1122191	C	-16
A1B1B7	Pden_1206	Urease accessory protein (UreF)	NtrY	-	Not detected	-	-	-	-
A1B1D8	Pden_1227	Uncharacterized protein	NtrY	-	Not detected	-	-	-	-
					Not detected	-	-	-	-
A1B1G0	Pden_1249	Binding-protein-dependent transport systems	NtrY	-	Not detected	-	-	-	-

		inner membrane component							
A1B1I5	Pden_1274	Uncharacterized protein	NtrY	-	**CT**GGGCGCTGTC**GC**	1233932	1233945	D	-105
					**CT**GGAGAGCGCG**GC**	1233985	1233998	D	-158
					**GA**GAGCGCGGCC**CG**	1233988	1234001	C	-161
A1B1K0	Pden_1289	Multisubunit potassium/proton antiporter, (PhaB)	NtrY	-	**CA**ATATTTTATC**GC**	1251847	1251860	D	-104
					**GT**TCGGGGTGTG**CG**	1251758	1251771	C	-15
					**GA**CGCAAAAATC**CG**	1251825	1251838	C	-82
A1B1L1	Pden_1300	Uncharacterized protein	NtrY	-	**CA**GCGGGGCAAC**GC**	1262736	1262749	D	-70
					**CA**TTCCTTGCAA**GC**	1262750	1262763	D	-56
A1B2F0	Pden_1594	Polyphosphate kinase	NtrY	-	**CT**TTGCCCTCTC**GC**	1574474	1574487	D	-13
A1B2Y8	Pden_1785	Helix-turn-helix domain protein	NtrY	-	**CT**GCGCGTCCCG**GC**	1776578	1776591	D	Pden_1783
A1B3X7	Pden_2129	Uncharacterized protein	NtrY	-	**GA**CGGGGGCGGC**CG**	2129834	2129847	C	Pden_2128
A1B4G4	Pden_2317	Uncharacterized protein	NtrY	-	**CA**TCCAGCGATT**GC**	2316365	2316378	D	-32
					**CA**AATCCGGCCG**GC**	2316389	2316402	D	-56
					**CT**TGAACATGCT**GC**	2316587	2316600	D	-254
					**CT**GGTCCAGCAT**GC**	2316608	2316621	D	-275
					**GT**TTGTCAAATC**CG**	2316383	2316396	C	-50
					**GT**CAAATCCGGC**CG**	2316387	2316400	C	-54
					**GT**TGGGGCGTTC**CG**	2316535	2316548	C	-202
					**GT**TCCGTTATGC**CG**	2316543	2316556	C	-210
					**GT**CCAGCATGCG**CG**	2316611	2316624	C	-278
					**GA**CTAAAATACG**CG**	2316511	2316524	C	-178
					**GA**TGAAGAATAC**CG**	2316630	2316643	C	-297
A1B4G7	Pden_2320	Uncharacterized protein	NtrY	-	Not detected	-	-	-	-
A1B4K8	Pden_2362	Coenzyme PQQ synthesis protein B	NtrY	-	**CA**CCGCATGAAT**GC**	2358351	2358364	D	-178
					**CA**CGGGTCGCGG**GC**	2358424	2358437	D	-251
					**CT**CAAGGTCACC**GC**	2358343	2358356	D	-170
					**GA**ATATCGCCGG**CG**	2358223	2358236	C	-50
					**GA**TTTCCTTCGG**CG**	2358299	2358312	C	-126
					**GA**TGGCACGGGT**CG**	2358419	2358432	C	-246
A1B4Q2	Pden_2408	Uncharacterized protein	NtrY	-	Not detected	-	-	-	-
A1B4T8	Pden_2445	Uncharacterized protein	NtrY	-	**CT**TTGCCCGGGG**GC**	2450878	2450891	D	-126
					**CT**GCGGCACCGG**GC**	2450903	2450916	D	-101
					**CT**TGCCGAACAG**GC**	2450986	2450999	D	-18
A1B4U2	Pden_2449	FHA domain containing protein	NtrY	-	Not detected	-	-	-	-
A1B575	Pden_2582	Uncharacterized protein	NtrY	-	**CA**CGGCTAATAG**GC**	2594642	2594655	D	-118

A1B5F1	Pden_2658	Major facilitator superfamily MFS_1	NtrY	-	**CA**CAGCTT GGCC**GC**	2668470	2668483	D	Pden_2657
A1B5M3	Pden_2733	Uncharacterized protein	NtrY	-	Not detected	-	-	-	-
A1B5M4	Pden_2734	Uncharacterized protein	NtrY	-	Not detected	-	-	-	-
A1B5X2	Pden_2832	Rieske (2Fe-2S) domain protein	NtrY	-	**CA**GCCGAA TGTC**GC**	2851921	2851934	D	-176
					**CT**GCCGTA ACTT**GC**	2851799	2851812	D	-54
					**CT**CCGTCC GGTC**GC**	2852031	2852044	D	-286
A1B6G6	Pden_3027	Alkyl hydroperoxide reductase (AhpD)	NtrY	-	**GA**CGCTTG CCGC**CG**	201716	201729	C	Pden_3026
A1B6R9	Pden_3132	Rhodanese domain protein	NtrY	-	**GT**CGGAAT GGAA**CG**	316380	316393	C	-25
					**GA**ACGCGA ACTT**CG**	316389	316402	C	-34
A1B6X4	Pden_3187	Uncharacterized protein	NtrY	-	**GA**CCGCGC CCAC**CG**	352722	352735	C	-172
					**GT**CGGTCG GCGA**CG**	352812	352825	C	-82
A1B712	Pden_3225	3-hydroxyacyl-CoA dehydrogenase	NtrY	-	**GT**TCGAGG CAAC**CG**	394680	394693	C	Pden_3228
A1B713	Pden_3226	Acetyl-CoA acetyltransferase	NtrY	-	**GT**TCGAGG CAAC**CG**	394680	394693	C	Pden_3228
A1B751	Pden_3267	Amidohydrolase 2	NtrY	-	**GA**CGAAA ACTGG**CG**	431605	431618	C	-5
A1B781	Pden_3297	Heavy metal translocating P-type ATPase	NtrY	-	Not detected	-	-	-	-
A1B7S2	Pden_3496	3-carboxy-*cis,cis*-muconate cycloisomerase	NtrY	-	Not detected	-	-	-	-
A1B7S6	Pden_3500	Transcriptional regulator, LysR family	NtrY	-	**GA**TTGGCT GCAA**CG**	646914	646927	C	-71
A1B7T9	Pden_3513	Transcriptional regulator, LysR family	NtrY	-	Not detected	-	-	-	-
A1B867	Pden_3644	Uncharacterized protein	NtrY	-	Not detected	-	-	-	-
A1B8A3	Pden_3680	GCN5-related *N*-acetyltransferase	NtrY	-	**CT**CCCTCC CCGC**GC**	828518	828531	D	-8
A1B8C2	Pden_3699	5-aminolevulinate synthase Heme A	NtrY	-	**GA**TTTCCG GGTG**CG**	847722	847735	C	-9
A1B8M9	Pden_3807	Lytic transglycosylase, catalytic	NtrY	-	**GA**AATCCT CTTG**CG**	943434	943447	C	-66
					**CT**TGCGGC GCCG**GC**	943442	943455	D	-58
					**GT**CTTTGG TTCC**CG**	943300	943313	C	-200
A1B8U2	Pden_3870	Cyclase/dehydrase	NtrY	-	**CT**GGATGC GGCG**GC**	1005041	1005054	D	Pden_3871
A1B8Z3	Pden_3921	Serine-glyoxylate aminotransferase apoenzyme	NtrY	-	**CT**TGTGGA CCAA**GC**	1057157	1057170	D	-16
					**CT**GTCCAT TGAT**GC**	1057180	1057193	D	-39
					**CT**CTTTTC CAAA**GC**	1057261	1057274	D	-120
					**GT**GGAATC AGAT**CG**	1057244	1057257	C	-103
					**GA**CCTAAA GTGT**CG**	1057231	1057244	C	-90
A1B941	Pden_3969	Uncharacterized protein	NtrY	-	Not detected	-	-	-	-
A1B9L3	Pden_4143	tRNA-specific adenosine deaminase	NtrY	-	**CT**GCGCAT CTCT**GC**	1299496	1299509	D	-85

					**CT**TGCGCG CAAG**GC**	1299560	1299573	D	-21
A1B9Q3	Pden_4183	7-carboxy-7-deazaguanine synthase	NtrY	-	**GT**AGGCTG CGAC**CG**	1341422	1341435	C	Pden_4185
A1BAJ5	Pden_4475	Uncharacterized protein	NtrY	-	**CT**CCTCAG GCTG**GC**	1691784	1691797	D	Pden_4477
A1BAU9	Pden_4580	NADPH-dependent FMN reductase	NtrY	-	**GA**GATTCA TATC**CG**	63178	63191	C	-69
					**CA**CTGGTA AGCG**GC**	63198	63211	D	-50
A1BB66	Pden_4699	Cytochrome B561	NtrY	-	**GT**GTTGGG CTTG**CG**	194500	194513	C	Pden_4700
A1BBB9	Pden_4752	Aldehyde dehydrogenase	NtrY	-	**GA**TGCGGC GGGA**CG**	243082	243095	C	Pden_4754
A1BBF3	Pden_4786	Phosphonate ABC transporter, periplasmic phosphonate-binding protein	NtrY	-	Not detected	-	-	-	-
A1BBI8	Pden_4821	Uncharacterized protein	NtrY	-	**GT**GGATCG ATGG**CG**	312182	312195	C	Pden_4816
A1BBM5	Pden_4859	4-hydroxyproline 2-epimerase	NtrY	-	Not detected	-	-	-	-
A1BBP0	Pden_4874	Polyamine-transporting ATPase	NtrY	-	Not detected	-	-	-	-
A1BBX3	Pden_4957	Transcriptional regulator, AsnC family	NtrY	-	**CA**CAGATT ACCG**GC**	463992	464005	D	-206
					**CA**AGATAT ATTC**GC**	464088	464101	D	-110
					**CT**GCTAGT CTGC**GC**	464151	464164	D	-47
					**GA**TGAATT CTGC**CG**	464013	464026	C	-185
					**GA**CTCGGA GTCC**CG**	464168	464181	C	-30
					**GA**GTCCCG GTCG**CG**	464174	464187	C	-24
A1BC07	Pden_4991	Alcohol dehydrogenase GroES domain protein	NtrY	-	Not detected	-	-	-	-
A1BC62	Pden_5046	Transcriptional regulator, MarR family	NtrY	-	**CA**AAAGAC ATAT**GC**	559309	559322	D	-101
					**CA**GAACAA CCAG**GC**	559368	559381	D	-161
					**GT**GCGGGT ATTG**CG**	559259	559272	C	-52
					**GT**CGGATA CTGT**CG**	559335	559348	C	-128
					**GT**CGAGCG CCTG**CG**	559345	559358	C	-138
					**GA**ATGATC CGCC**CG**	559224	559237	C	-17
					**GA**CTGCCA GAGT**CG**	559325	559338	C	-118
					**GA**TACTGT CGAG**CG**	559339	559352	C	-132
					**GA**GCGCCT GCGG**CG**	559348	559361	C	-141
A1B2P3*	Pden_1690	Transcriptional regulator, BadM/Rrf2family (RirA)	NtrY	-	**CA**AGGTTG CGCG**GC**	1672611	1672624	D	-98
					**CT**GCAAGG TTGC**GC**	1672608	1672621	D	-101

(*) Detected only in two or one biological samples or displayed a high *p*-value. [1]Protein annotated from UniProt (UP000000361). [2]Genes annotated from GeneBank (T00440). [3]Fold change calculated as the ratio of protein expression in the wild-type/NtrY strain. [4]Orientation of the NtrX binding sequence (C: complementary strand, D:

direct strand), [5] Position (from the start codon) of the of predicted NtrX binding boxes in the promoter regions of genes encoding affected proteins in this study. In several promoter regions NtrX binding boxes could not be detected, but they were found in the promoter regions of adjacent genes.

Table S2. *Paracoccus denitrificans* PD1222 iron-responsive regulator homologs in α-proteobacteria.

Bacterial strains*	Irr	MUR	FUR	RirA	IscR
Rhizobium leguminosarum bv. viciae	A1B1H1/<br>Pden_1260<br>Id.: 42.0 %<br>Cov.: 71 %				
Rhizobium etli CFN 42	A1B1H1/<br>Pden_1260<br>Id.: 42.86 %<br>Cov.: 65 %				
Rhizobium tropici CIAT 899		A1B1H1/<br>Pden_1260<br>Id.: 38.35 %<br>Cov.: 93 %			
Bradyrhizobiaceae bacterium SG-6C		A1B1H1/<br>Pden_1260<br>Id.: 45.19 %<br>Cov.: 83 %			
Rhizobium leguminosarum bv. viciae 3841		A1B1H1/<br>Pden_1260<br>Id.: 42.64 %<br>Cov.: 90 %			
Bradyrhizobium japonicum<br>(“*Bradyrhizobium diazoefficiens*”)			A1B1H1/<br>Pden_1260<br>Id.: 43.94 %<br>Cov.: 87 %		
Zymomonas mobilis			A1B1H1/<br>Pden_1260<br>Id.: 49.15 %<br>Cov.: 82 %		
Rhizobium etli				A1B2P3/<br>Pden_1690<br>Id.: 40.94 %<br>Cov.: 93 %<br><br>A1B6G3/<br>Pden_3024<br>Id.: 40.00 %<br>Cov.: 81 %<br><br>A1B9P2/<br>Pden_4172<br>Id.: 40.60 %<br>Cov.: 82 %	
Sinorhizobium meliloti SM11				A1B2P3/<br>Pden_1690<br>Id.: 41.18 %<br>Cov.: 99 %<br><br>A1B9P2/<br>Pden_4172<br>Id.: 42.75 %<br>Cov.: 85 %<br><br>A1B6G3/<br>Pden_3024<br>Id.: 41.86 %<br>Cov.: 83 %	
Agrobacterium tumefaciens<br>(“*Rhizobium radiobacter*”)				A1B9P2/<br>Pden_4172<br>Id.: 38.17 %<br>Cov.: 83 %<br><br>A1B6G3/<br>Pden_3024	

				Id.: 37.21 %<br>Cov.: 82 %	
Brucella melitensis				A1B2P3/<br>Pden_1690<br>Id.: 41.84 %<br>Cov.: 92 %	
Rhodospirillum rubrum ATCC 11170					A1B698/<br>Pden_2958<br>Id.: 42.48 %<br>Cov.: 99 %
Caulobacter vibrioides CB15					A1B698/<br>Pden_2958<br>Id.: 46.90 %<br>Cov.: 91 %
Zymomonas mobilis subsp. mobilis ZM4 (= ATCC 31821)					A1B698/<br>Pden_2958<br>Id.: 36.67 %<br>Cov.: 65 %

(*) Bacterial strains were selected according to a previous study [22]. The protein sequenced were aligned with the Blastp from NCBI (https://blast.ncbi.nlm.nih.gov/Blast.cgi), yielding the identity (Id.) and coverage (Cov.).

Table S3. Oligonucleotides used in the qRT-PCR analysis of *P. denitrificans* PD1222.

Primer name*	Name	Sequence (5'-3')
Pden_0893F	Cytochrome *c* peroxidase (Ccp)	GTCAGCTTCGACAACTTCGC
Pden_0893R		TGCAGCCGGTCTCGATAAAG
Pden_1260F	Manganese uptake regulator, Fur family (Mur/Irr)	CTGGCGACGGTTTATCGCAC
Pden_1260R		GCCGGTCTCGACATCGATCA
Pden_1355F	Methionine synthase (B_{12}-independent)	CAATGCCATCGAAACACCCG
Pden_1355R		CCCGCCAGAATTTCGTTTCG
Pden_1690F	Transcriptional regulator, BadM/Rrf2family (RirA)	CTTGTGCGCCATACCGAGGA
Pden_1690R		CAGGCTGTAGCCGTCCAGAA
Pden_1848F	Cytochrome *c* oxidase, cbb_3-type, subunit I	CCATGCTGCATATCGTCAAC
Pden_1848R		CATACCACCATTGCGTCATC
Pden_1850F	Putative transcriptional regulator, Crp/Fnr family (FnrP)	CTTGTGCTGTGCTGCTTTCG
Pden_1850R		CAAGCTCGTCCAGCGTCATC
Pden_1937F	Cytochrome c_{550}	CGAGGCCGACCTGATCGAAT
Pden_1937R		GCCCTTGTCGTCGGTCATCT
Pden_2046F	TonB-dependent receptor	CCAGATCACCGCCGAGGATT
Pden_2046R		GCGAGAAGCTGAGCTCGGTA
Pden_2305F	Ubiquinol-cytochrome *c* reductase iron-sulfur subunit	AAAACCGCACGATGGACGAG
Pden_2305R		AATTCGGCGACGGGGATATG
Pden_2484F	Nitric oxide reductase subunit C (NorC)	ACCAAGAACATGGCCCGGAA
Pden_2484R		CCTTGGTGCGGGCGTAGATA
Pden_2486F	Protein (NirI)	TCTATTGCCGCTACCTGTGC
Pden_2486R		GACAGTGCATTTCGTCTCGC
Pden_2487F	Nitrite reductase (NirS)	TCCGACGCCCAATACAACGA
Pden_2487R		TGCAGGTAGTCGAAGCCCAG
Pden_2610F	TonB-dependent receptor	ATGGCCAGGAGAACGTCGAG
Pden_2610R		CCGGAAACGGTGATGGAGGA
Pden_2832F	Rieske (2Fe-2S) domain protein	TCTGGTATCGCGAATGGCTC
Pden_2832R		ATCACCACCGGATATTCGCC
Pden_2958F	Transcriptional regulator, BadM/Rrf2 family (IscR)	ACATCTCGCTGCCCTATCTG
Pden_2958R		GATATGCAATGCGCTGACGG
Pden_3027F	Alkyl hydroperoxide reductase (AhpD)	AACGAGCAGATCATGGAGGC

Pden_3027R		TCTCATCCAGAACCGCAAGC
Pden_4044F	NnrS family protein	TCTGCCTTATTCACGCCTCG
Pden_4044R		GCATGGCGGAAATCCCAAAG
Pden_4127F	Two component, σ^{54} specific, transcriptional regulator Fis family (NtrX)	TCTATTTCGACGAGGTGGCG
Pden_4127R		TTCAGCCGGTCGTAGAGTTC
Pden_4128F	PAS/PAC sensor signal transduction histidine kinase (NtrY)	CGGTGAATATCGGGCTGGAGGGCTGG
Pden_4128R		TGGCGTCGTTGGTCAAGTCTCGGCG
Pden_4139F	Putative ferric uptake regulator, Fur family	CTTCGGAATCCCCGCCTGAC
Pden_4139R		CCTTCGGTCCTTGCCTGCTC
Pden_4213F	Acetyl-coenzyme A synthetase (AcCoA synthetase)	AAGCCCGACCTGATCCAATG
Pden_4213R		TCGATGAGTTGGTCCACGAC
Pden_4220F	FMN-binding domain protein (NosR)	TGTCGGTCGTGAACCTGATG
Pden_4220R		ACCTGGTTGGTCAGCTCTTG
Pden_4221F	Uncharacterized protein (NosC)	CCTTGCAGGAGGTCGCCATT
Pden_4221R		GGGTGTTCATTGCCTTCGGC
Pden_4222F	Pseudoazurin	TGACCGAGCCCGGACTTTAC
Pden_4222R		GCCTTCTTGGGCATCTTCGC
Pden_4230F	NnrS family protein	CTGCTGGCATTGGGTTTCTG
Pden_4230R		CCGATGAGCGCCAGATATAG
Pden_4237F	Nitrate/nitrite transporter (NarK)	TCGTCTGGGTGCCCTTCATC
Pden_4237R		AGCAGGCACATCACCCAGTT
Pden_4453F	Major facilitator superfamily MFS_1 (NasA)	CAGATCCGCGAGCAGCTTTC
Pden_4453R		ATCGTATAGACCAGCCGCCC
Pden_4455F	Response regulator receiver and ANTAR domain protein (NasT)	TCGTCATCGAATCCGACCCG
Pden_4455R		GAGGCCGGTTTCTTCCGAGA
Pden_4719F	Periplasmic nitrate reductase subunit (NapE)	ATCGTCCCAAGCACCAGAAG
Pden_4719R		AGACGATGAATCCGTAGCCG
Pden_5107F	Cytochrome ba3 quinol oxidase subunit 1	CGTGTTTTCCGAAGTCACCT
Pden_5107R		GCCCATGGTGAAGAAGTGAT

(*) F: forward, R: reverse.

Supplementary Figure legends

Figure S1. *P. denitrificans* gene clusters with NtrX targets genes. (**A**) Pden_4124, RNA-binding protein Hfq; Pden_4125, cation transporter; Pden_4126, TrkA-N domain protein; Pden_4127, two-component system, sigma54 specific, transcriptional regulator NtrX; Pden_4128, PAS/PAC sensor signal transduction histidine kinase NtrY; Pden_4129, two component, sigma54 specific, transcriptional regulator NtrC; Pden_4130, signal transduction histidine kinase, nitrogen specific NtrB; Pden_4131, tRNA-U20-dihydrouridine synthase; Pden_4132, L-serine ammonia-lyase. (**B**) Pden_4230, NnrS family protein; Pden_4231, conserved hypothetical protein; Pden_4232, PpiC-type peptidyl-prolyl cis-trans isomerase; Pden_4233, respiratory nitrate reductase gamma subunit NarI; Pden_4234, respiratory nitrate reductase chaperone NarJ; Pden_4235, respiratory nitrate reductase beta subunit NarH; Pden_4236, respiratory nitrate reductase alpha subunit apoprotein NarG; Pden_4237, nitrate/nitrite transporter NarK; Pden_4238, putative transcriptional regulator, Crp/Fnr family NarR; Pden_4239, DNA-O6-methylguanine--protein-cysteine S-methyltransferase; Pden_4240, FAD-binding 9, siderophore-interacting domain protein; Pden_4241, drug resistance transporter, Bcr/CflA subfamily; Pden_4242, conserved hypothetical protein. Pden_2478, putative transcriptional regulator, Crp/Fnr family NNR; Pden_2479, hypothetical protein NorF; Pden_2480, nitric oxide reductase NorE protein; Pden_2481, nitric oxide reductase NorD protein; Pden_2482, nitric oxide reductase NorQ protein; Pden_2483, nitric oxide reductase, NorB subunit apoprotein; Pden_2484, nitric oxide reductase, NorC subunit apoprotein; Pden_2485, FAD:protein FMN transferase; Pden_2486, NosR/NirI family transcriptional regulator, nitrite reductase regulator; Pden_2487, nitrite reductase NirS; Pden_2488, uroporphyrinogen-III C-methyltransferase; Pden_2489, cytochrome c55X.

Pden_4213, acetyl-coenzyme A synthetase; Pden_4214, FAD:protein FMN transferase NosX; Pden_4215, NosL family protein; Pden_4216, nitrous oxide maturation protein NosY; Pden_4217, ABC transporter related protein NosF; Pden_4218, periplasmic copper-binding protein NosD; Pden_4219, nitrous oxide reductase apoprotein NosZ; Pden_4220, NosR/NirI family transcriptional regulator, nitrous oxide reductase regulator; Pden_4221, hypothetical protein; Pden_4222, pseudoazurin; Pden_4223, conserved hypothetical protein. (**C**) Pden_4456, MOSC domain containing protein; Pden_4455, response regulator receiver and ANTAR domain protein NasT; Pden_4454, two-component system, oxyanion-binding sensor NasS; Pden_4453, MFS transporter, NNP family, nitrate/nitrite transporter NasA; Pden_4452, assimilatory nitrite reductase (NAD(P)H) large subunit precursor NasB; Pden_4451, assimilatory nitrite reductase (NAD(P)H) small subunit NasG; Pden_4450, formate/nitrite transporter NasH; Pden_4449, assimilatory nitrate reductase (NADH) alpha subunit apoprotein NasC; Pden_4448, iron(III) transport system ATP-binding protein; Pden_4447, iron(III) transport system substrate-binding protein. Pden_4717, TRAP dicarboxylate transporter, DctM subunit; Pden_4718, hypothetical protein; Pden_4719, periplasmic nitrate reductase subunit NapE; Pden_4720, periplasmic nitrate reductase chaperone NapD; Pden_4721, periplasmic nitrate reductase subunit NapA apoprotein; Pden_4722, periplasmic nitrate reductase subunit NapB; Pden_4723, periplasmic nitrate reductase subunit NapC.

Figure S2. PCA analysis of the quantitative proteomic data. PCA analysis of *P. denitrificans* PD1222 wild-type strain and NtrY mutant proteomes under denitrifying conditions. The analysis was carried out in Perseus (version 1.6.2.3). Only LFQ intensity values of differentially expressed proteins with respect to the reference proteome (wild-type strain) were used. Missing values were imputed with the value of the mean of the

five lowest LFQ normalized intensity values of the whole experiment. Resulting data were normalized by Z-score using the mean without grouping.

Figure S3. Hierarchical clustering of *P. denitrificans* PD1222 proteomes obtained by LC-MS/MS analyses of the wild-type strain and NtrY mutant. Clustering approach was carried out in Perseus (version 1.6.2.3). Values previously assigned were used for a hierarchical cluster by using Euclidean distance. Eleven clusters representing different expression profiles were defined by using a distance threshold of 3.43253755569458.

Figure S4. Volcano plot of the proteomic data. PCA analysis of *P. denitrifcans* PD1222 wild-type strain and NtrY mutant proteomes under denitrifying conditions.

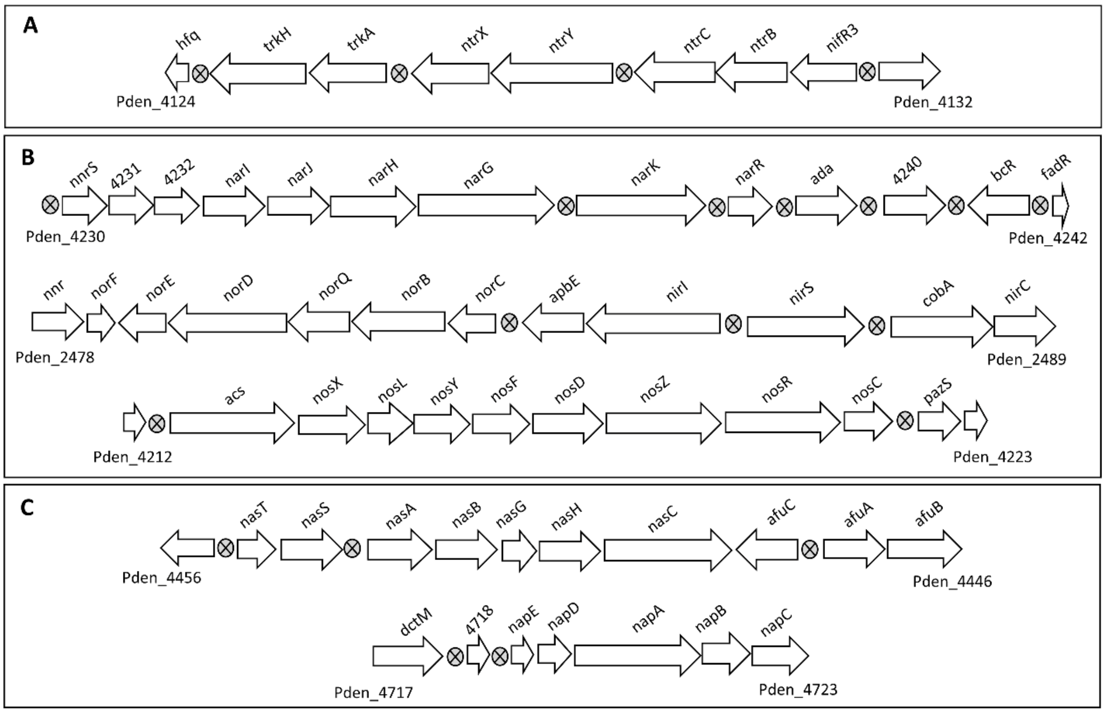
A
hfq
trkH
trkA
ntrX
ntrY
ntrC
ntrB
nifR3
Pden_4124
Pden_4132
B
nnrS
4231
4232
narI
narJ
narH
narG
narK
narR
ada
4240
bcR
fadR
Pden_4230
Pden_4242
nnr
norF
norE
norD
norQ
norB
norC
apbE
nirI
nirS
cobA
nirC
Pden_2478
Pden_2489
acs
nosX
nosL
nosY
nosF
nosD
nosZ
nosR
nosC
pazS
Pden_4212
Pden_4223
C
nasT
nasS
nasA
nasB
nasG
nasH
nasC
afuC
afuA
afuB
Pden_4456
Pden_4446
dctM
4718
napE
napD
napA
napB
napC
Pden_4717
Pden_4723

Figure S1

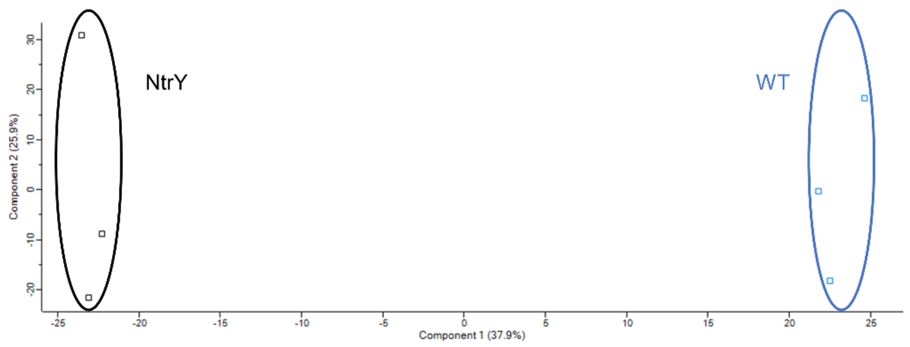

Figure S2

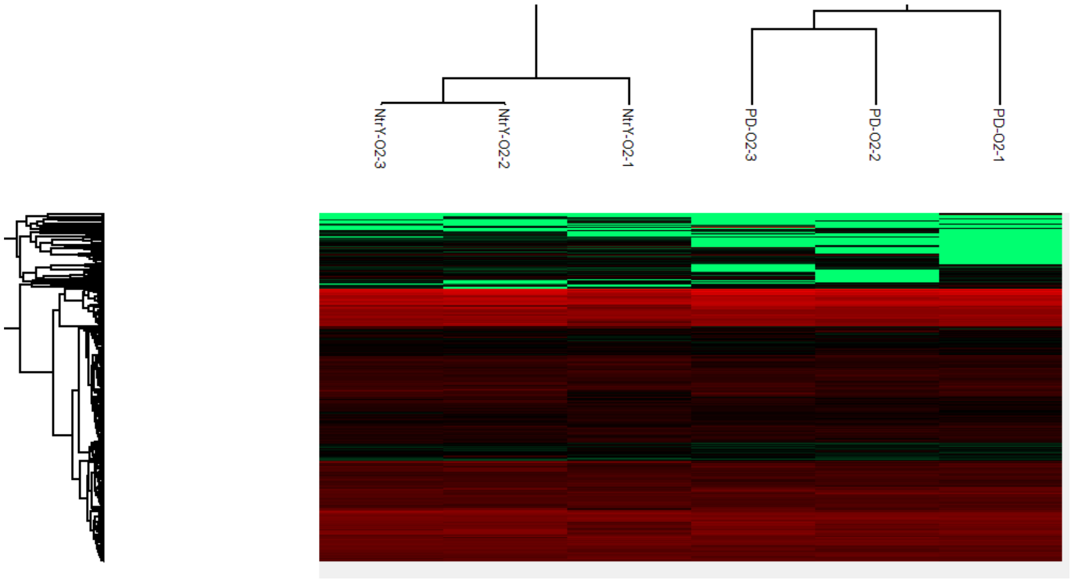

Figure S3

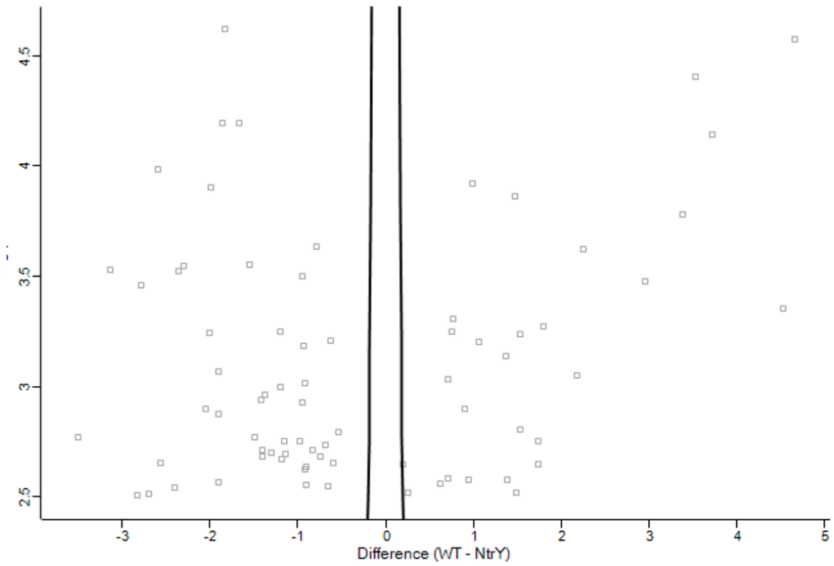

Figure S4
